# Zein-Induced Enhancement of the Thermal Stability of Anthocyanins Extracted from Agricultural Residues of Red Corn (*Zea mays*)

**DOI:** 10.3390/molecules31142499

**Published:** 2026-07-17

**Authors:** Saúl González-Cuna, Cristian Jiménez-Martínez, Ana Elena Cedillo-Olivos, Yair Cruz-Narváez, Luis Jorge Corzo-Ríos, Liliana Alamilla-Beltrán

**Affiliations:** 1 Escuela Nacional de Ciencias Biológicas, Instituto Politécnico Nacional, Av. Wilfrido Massieu 399, Nueva Industrial Vallejo, Gustavo A. Madero, Mexico City 07700, Mexico; sgonzalezc@ipn.mx (S.G.-C.); acedilloo1700@alumno.ipn.mx (A.E.C.-O.); 2 Laboratorio de Posgrado e Investigación de Operaciones Unitarias, Escuela Superior de Ingeniería Química e Industrias Extractivas, Instituto Politécnico Nacional, Zacatenco, Unidad Profesional Adolfo López Mateos, Colonia Lindavista, Mexico City 07738, Mexico; ycruzn@ipn.mx; 3 Unidad Profesional Interdisciplinaria de Biotecnología, Instituto Politécnico Nacional, Av. Acueducto S/N, Barrio La Laguna, Col. La Laguna Ticoman, Mexico City 07340, Mexico; lcorzo@ipn.mx

**Keywords:** anthocyanins, zein, thermal stability, red corn chaff

## Abstract

The stability of anthocyanins is a key factor limiting their use as functional natural colorants. This study evaluated the protective effect of zein on anthocyanins extracted from red corn (*Zea mays*) chaff, which is an underutilized agro-industrial byproduct. The extraction of anthocyanins was optimized using a Box–Behnken design, employing a 55.5% (*v*/*v*) aqueous ethanol solution at 45.21 °C and a solid-to-solvent ratio of 1.86 mg/mL (*w*/*v*). The resulting extract was rich in acylated anthocyanins derived from cyanidin, pelargonidin, and malvidin, which were identified using electrospray ionization Fourier transform ion cyclotron resonance mass spectrometry (FIA-ESI-FTICR-MS). The interaction between zein and the anthocyanins was found to be thermodynamically spontaneous (indicated by a negative ΔG), endothermic (ΔH = 19.8 kJ mol^−1^), and primarily driven by hydrophobic interactions (ΔS = 84.5 J mol^−1^ K^−1^). The addition of zein significantly decreased the degradation rate constant of the anthocyanins and increased their half-life, especially at pH 4 and storage temperatures of 4 °C and 20 °C. Moreover, the activation energy (E_a_) for the zein–anthocyanin complex formation was lower (ranging from 63.64 to 47.75 kJ mol^−1^) than that of the extract without zein (ranging from 59.36 to 74.71 kJ mol^−1^), indicating that the zein complex has lower thermal sensitivity. Additionally, zein enhanced the red hues of the extract (increasing a* to 62.66) and preserved its antioxidant capacity, which even increased by up to 3.5 times under accelerated degradation conditions. Zein serves as an accessible and effective plant-based alternative for stabilizing anthocyanins in acidic environments, with potential applications in the development of functional natural colorants for the food industry.

## 1. Introduction

Chaff is an agricultural residue generated during the processing of various grain crops, such as corn, wheat, and oats, among other cereals [1]. In the case of corn, this material is composed of husk fragments, cob (cob or rachis), shredded leaves, and small parts of the plant’s stalk. Its main characteristic is its low bulk density, which makes it difficult to handle, as the fine particles that constitute it tend to become easily airborne, making it unattractive for reuse in any production process [2].

Corn chaff is primarily composed of biopolymers such as cellulose (33.7–35%, *w*/*w*), hemicellulose (16.8–31.9%, *w*/*w*), and lignin (6.1–7%, *w*/*w*). The low digestibility of these compounds in humans has traditionally limited their applications to the production of fermentable carbohydrates via hydrolysis, thereby generating bioethanol and biodiesel. In addition, corn chaff has been used as a substrate for microbial growth and as a partial substitute for plant-derived materials in silage formulations for animal feed production [3,4,5,6].

However, chaff from pigmented corn represents a potential source of bioactive molecules, particularly anthocyanins, which are responsible for the characteristic coloration of different corn varieties. Anthocyanins are polyphenols belonging to the flavonoid family. Structurally, they consist of a flavylium ion (2-phenylchromenyl) substituted with hydroxyl, methyl, or methoxyl groups, called anthocyanidin, to which a monosaccharide is attached via a glycosidic bond. In this structure, electron delocalization through σ and π bonds is responsible for the absorption and emission of photons that generate color perception [7]. In pigmented maize, anthocyanins such as cyanidin-3-*O*-glucoside, peonidin-3-*O*-glucoside, and pelargonidin-3-*O*-glucoside have been identified, as well as other forms acylated with malonic acid, such as malonyl-glucoside derivatives [8].

These molecules have been attributed to various biological activities, including antioxidant, anti-inflammatory, anticancer, and neuroprotective properties. Consequently, the search for sources of anthocyanins has gained importance, with the aim of extracting them and incorporating them as natural colorants in food to leverage their beneficial health effects [9,10,11,12].

Nonetheless, the application of anthocyanins as food colorants is limited by their pronounced instability under various physical and chemical conditions, including temperature fluctuations, pH changes, and exposure to oxygen, light, and oxidizing agents. This instability has restricted their incorporation into a wide range of food matrices [13]. Numerous strategies have been developed to enhance anthocyanin stability, including microencapsulation, copigmentation, self-association, and the incorporation of acidic matrices [14,15].

Among these mechanisms, interactions with other molecules represent an important factor in the stabilization of anthocyanins. In wine, for example, it is considered one of the main mechanisms responsible for color preservation. Furthermore, reactions between anthocyanins and copigments, particularly tannins, can lead to the formation of more stable-colored compounds known as pyranoanthocyanins [16]. These pigments can persist throughout wine fermentation and aging, contributing to color stability over time despite exposure to adverse storage conditions, including light and temperature [17].

In this context, several classes of molecules have been reported to act as copigments of anthocyanins, including organic acids [18], other flavonoids such as rutin and quercetin [19], proteins [20], glycoproteins such as gum arabic [21], and amino acids [22]. Among these, interactions with proteins have attracted particular attention due to their protective effects on anthocyanin stability. For instance, both native and denatured whey proteins have been shown to delay anthocyanin hydrolysis under elevated temperatures and in the presence of ascorbic acid. Likewise, α- and β-casein, β-lactoglobulin, and bovine serum albumin have been reported to stabilize anthocyanins and protect them against thermal degradation [23,24]. Recent research indicates that whey protein isolate (WPI) significantly protects anthocyanins from blue honeysuckle (*Lonicera caerulea*) through strong hydrophobic interactions. This protection increases anthocyanin retention by 4.1-fold under light and storage conditions and by 3.92-fold after simulated digestion [25]. These findings highlight the critical role of hydrophobic interactions as the main mechanism for stabilizing the protein–anthocyanin complex.

However, the interaction between anthocyanins and plant-derived proteins has received limited attention in scientific literature. Interactions between anthocyanins from black rice and proteins isolated from rice, as well as soy protein isolate, have been previously reported [26,27]. In a recent study, Eyiz et al. [28] evaluated the copigmentation of anthocyanins derived from *Hibiscus sabdariffa* when combined with protein isolates from pea, rice, gluten, and whey. Their findings showed that rice and whey proteins enhance the thermal stability of anthocyanins, resulting in activation energies of up to 47.98 kJ mol^−1^. Additionally, microencapsulation research has demonstrated that mixtures of pea and rice proteins, combined with fructooligosaccharides, can extend the half-life of anthocyanins to as long as 364.8 days, thereby improving their bioaccessibility during digestion [29]. The mechanisms governing protein–anthocyanin association involve hydrophobic interactions between phenolic compounds and aromatic amino acid residues; hydrogen bond formation between amino (NH_2_), sulfhydryl (SH), hydroxyl (OH), and carboxyl (COOH) groups of amino acid residues and the delocalized electrons of anthocyanins; van der Waals forces; and, to a lesser extent, ionic interactions [30]. Additionally, recent reviews [31] have systematized knowledge on the degradation mechanisms of anthocyanins (hydration, ring opening, oxidative cleavage, and deglycosylation), as well as stabilization strategies via intra- and intermolecular copigmentation and encapsulation, thereby providing selection criteria for different food matrices.

Recent studies have shown that zein, a prolamin derived from maize, can effectively stabilize anthocyanins through noncovalent interactions, primarily hydrophobic forces and hydrogen bonds [32]. In their research, Tian et al. [33] employed a molecular docking screening approach to identify anthocyanin-binding proteins. Their findings showed that zein–anthocyanin complexes exhibit flexible structural adaptations, significantly improving thermal stability in slightly acidic pH ranges (4.0–6.0). Similarly, Li et al. [34] developed zein nanoparticles modified with phenolic acids and lecithin, which enhanced the stability of anthocyanins against thermal and gastrointestinal degradation. This modification achieved a remarkable 1.9-fold increase in cellular transport efficiency through interactions with specific transporters. Moreover, co-encapsulating anthocyanins with gallic acid in zein–gum arabic nanoparticles resulted in retention rates of up to 74.81% after 25 days of storage [35]. This demonstrates significant progress in the development of controlled-release systems for these pigments.

Zein is the main storage protein found in maize (*Zea mays*), belonging to the prolamin group. It comprises 25–30% proline and glutamine among its amino acid residues [36]. The molecular weight of zein varies between 19 and 44 kDa, depending on the subunit (α, β, γ, or δ), with α-zein (19 and 22 kDa) being the predominant fraction, accounting for approximately 70 to 85% of the total [37]. Structurally, zein is characterized by a high content of hydrophobic amino acids, such as leucine, alanine, phenylalanine, isoleucine, valine, and proline. This composition promotes the formation of hydrophobic domains in its native conformation, resulting in very limited solubility in water but high solubility in hydroalcoholic mixtures (60–95% ethanol) and alkaline solutions (pH > 11) [38]. Zein’s isoelectric point is estimated at pH 6.2; below this pH, the protein carries a net positive charge and exhibits increased solubility and an extended conformation, whereas above it, it tends to aggregate and precipitate [32].

From a functional perspective, zein is widely used in the food and pharmaceutical industries for creating edible films, protective coatings, and encapsulation systems. Its ability to form hydrophobic matrices serves as an effective physical barrier against moisture and oxygen [37]. In terms of stabilizing bioactive compounds, zein effectively acts as a carrier for polyphenols and other hydrophobic compounds through non-covalent interactions primarily driven by hydrophobic forces, hydrogen bonds, and, to a lesser extent, electrostatic interactions [30,32].

The structure of zein, which is rich in α-helices (approximately 50–60% of its secondary structure), gives it an elongated, flexible shape, exposing hydrophobic regions on its surface. This feature facilitates interactions with nonpolar or amphiphilic molecules, such as anthocyanins [38]. Additionally, the presence of aromatic residues, including phenylalanine, tyrosine, and tryptophan, enables π-π stacking interactions with the delocalized π systems of flavonoids, thereby forming stable complexes that protect anthocyanins from thermal and oxidative degradation [30].

Recently, the application of zein as an anthocyanin stabilizer in food systems has been examined. Research shows that its ability to form complexes with these pigments depends critically on factors such as pH, temperature, and the protein-to-anthocyanin molar ratio [32]. Despite these advancements, our understanding of the molecular mechanisms governing zein–anthocyanin interactions and their effects on the thermal and colorimetric stability of extracts remains limited, particularly concerning agro-industrial byproducts such as pigmented corn cobs.

The present work focuses on the effect of zein, a plant protein that remains largely unexplored in the context of protein–anthocyanin interactions, on the stability of phenolic compounds, particularly anthocyanins, extracted from the agro-industrial residue of a red maize landrace. Specifically, the stability of these compounds was evaluated under different pH conditions and storage temperatures.

## 2. Results and Discussion

### 2.1. Optimization of the Extraction of Anthocyanins

**Figure 1:**
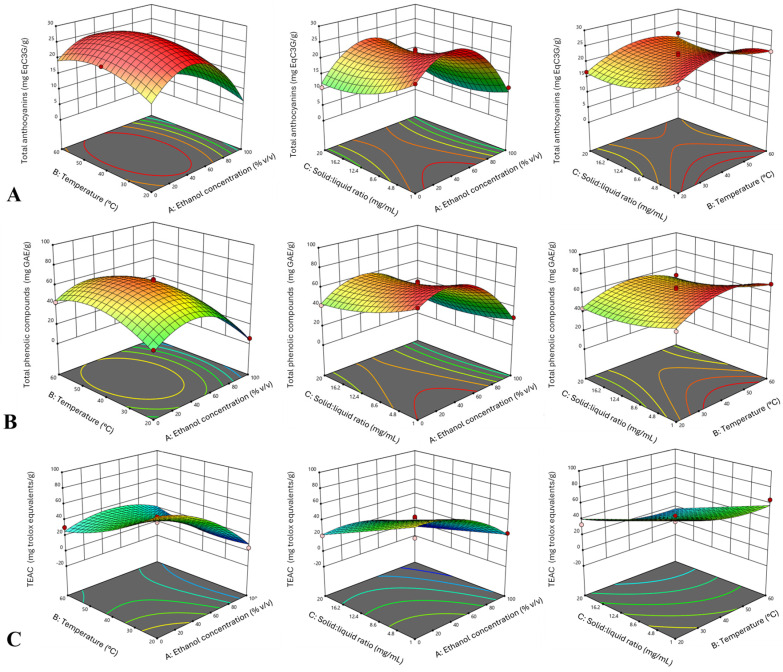
Response surfaces show the effect of temperature and ethanol concentration on the extraction of bioactive compounds from red corn chaff. (**A**) Total anthocyanins (mg C3GE g^−1^ of dry sample), (**B**) total phenolic compounds (mg GAE g^−1^ of dry sample), and (**C**) antioxidant capacity (mg Trolox equivalents g^−1^ of dry sample). The solid-to-solvent ratio was kept constant at its average value (10:1, *w*/*v*).

The concentrations of total phenolic compounds (mg GAE g^−1^ dry sample) and total anthocyanins (mg C3GE g^−1^ dry sample) are presented in Figure 1. The results indicate that both temperature and ethanol concentration significantly influenced the recovery of these compound groups. For total anthocyanins (Figure 1A) and total phenolic compounds (Figure 1B), a quadratic model provided the best fit to the experimental data, with coefficients of determination (R^2^) of 0.9825 and 0.9918 and adjusted R^2^ values of 0.9510 and 0.9764, respectively. In contrast, antioxidant capacity (Figure 1C) was best described by a linear model, with an R^2^ value of 0.9369 and an adjusted R^2^ of 0.82. In all cases, the lack-of-fit test was not statistically significant (*p* > 0.05), indicating that the models provided an adequate predictive performance.

It is important to note that the proposed linear model for antioxidant capacity, as measured by the DPPH assay, had a lower adjusted coefficient of determination (adjusted R^2^ = 0.82) compared to the quadratic models for total phenols and total anthocyanins, which had adjusted R^2^ values greater than 0.95. Despite this difference, the linear model’s R^2^ value is still acceptable for process optimization studies involving complex plant matrices.

This disparity suggests that antioxidant activity, as determined by the DPPH assay, may be influenced by experimental factors in a nonlinear way or by interactions among bioactive compounds that the linear model does not fully capture. Additionally, the inherent variability of the DPPH assay and the potential contribution of non-phenolic compounds with antioxidant properties could account for some of the observed variability.

Nonetheless, the model was statistically significant (*p* < 0.05), and the lack-of-fit test yielded a non-significant result (*p* > 0.05), which supports its predictive validity within the range of experiments conducted. This observation underscores the need to account for matrix complexity and the diversity of compounds present when interpreting the antioxidant capacity of agricultural by-product extracts.

The highest concentrations of phenolic compounds and anthocyanins were obtained with ethanol–water mixtures containing 40–60% (*v*/*v*) ethanol, particularly for anthocyanins (Figure 1A). This finding is consistent with previous reports showing that the highest anthocyanin recovery from berries was achieved at ethanol concentrations near 60% (*v*/*v*) [39]. The presence of water in the extraction solvent promotes hydration of the plant matrix, thereby facilitating the release of phenolic compounds. Meanwhile, ethanol enhances the solubility of anthocyanins and other polyphenols that are intermediate in polarity.

Temperature had a significant impact on extraction efficiency (*p* < 0.05). As the temperature increased, extraction efficiency improved due to enhanced diffusion and reduced viscosity, while the endothermic dissolution process raised the equilibrium solubility of phenolic compounds [40]. However, when the temperature reached or exceeded 60 °C, anthocyanin concentrations began to decline due to thermal degradation, including processes such as glycosidic bond hydrolysis, pyrylium ring opening, and chalcone formation [41]. The optimal extraction temperature was approximately 45 °C, as this level balanced improved extraction with the risk of thermal degradation.

In contrast, although the solid-to-solvent ratio also significantly affected the concentrations of the extracted compounds (*p* < 0.05), it had the smallest effect among the factors evaluated. The highest concentrations of phenolic compounds and anthocyanins were observed at low solid-to-solvent ratios (1:1, *w*/*v*), corresponding to a larger volume of solvent per unit mass of solid. This trend can be explained by the fact that increasing the solvent volume enhances the concentration gradient between the plant matrix and the liquid phase. This enhancement facilitates the diffusion of target compounds from the matrix into the solvent and accelerates mass transfer. However, it is important to note that the solvent volume does not affect the thermodynamic solubility of anthocyanins; rather, it influences the driving force for diffusion, as described by Fick’s law. A larger solvent volume creates a steeper concentration gradient, which promotes faster diffusion of solutes from the solid matrix into the bulk solution until equilibrium is reached. Consequently, this increases the overall extraction yield within a given time [40]. This principle has been extensively documented in the literature on solid–liquid extraction of phenolic compounds and anthocyanins [40].

A key finding was that radical-scavenging activity (Figure 1C) did not correlate directly with anthocyanin concentration in the extracts. The highest antioxidant activity was observed in aqueous extracts obtained at low temperatures, particularly in the region of Figure 1C corresponding to low ethanol concentrations (20–30% *v*/*v*). These results indicate that compounds preferentially extracted with water, such as organic acids, simple phenolic acids, and other hydrophilic constituents, exhibit a greater ability to inhibit the DPPH radical than compounds solubilized in hydroalcoholic mixtures. These molecules may act as highly effective electron- or hydrogen-donating agents, potentially exceeding the antioxidant effects typically attributed to anthocyanins and total phenolic compounds (determined by the Folin–Ciocalteu method, which estimates the overall reducing capacity of phenolic compounds and other reductants present in the extract). Similar observations have been reported in plant matrices, where non-anthocyanin constituents contribute substantially to the overall antioxidant activity [42].

The optimal extraction conditions for maximizing anthocyanin concentration were an extraction temperature of 45.21 °C, an ethanol concentration of 55.56% (*v*/*v*), and a solid-to-solvent ratio of 1.86 mg/mL. Under these conditions, the model predicted a maximum anthocyanin concentration of 25.48 mg C3GE g^−1^ dry sample. Experimental validation yielded an anthocyanin concentration of 23.29 mg C3GE g^−1^ dry sample, corresponding to a model predictive accuracy of 90.6%. This optimized extract was subsequently selected for phytochemical characterization and stability studies in liquid systems.

### 2.2. Phytochemical Study of Red Corn Chaff Extract

Table 1 presents the metabolomic profile of the optimized red maize chaff extract, obtained by flow injection coupled with electrospray ionization Fourier transform ion cyclotron resonance mass spectrometry (FIA-ESI-FTICR-MS).

The analysis revealed a rich and diverse composition of phenolic compounds, dominated by flavonols such as kaempferol, quercitrin, and isorhamnetin, together with their glycosylated derivatives. In addition, flavones such as chrysoeriol and flavanones such as eriodictyol were identified, along with a wide variety of anthocyanins derived primarily from cyanidin, pelargonidin, and malvidin. This metabolomic profile is consistent with previous reports on pigmented maize varieties and other cereal crops [43,44].

The relative abundances shown in Table 1 represent ESI signal intensities rather than absolute concentrations, as ionization efficiency is dependent on structure. While these values facilitate qualitative comparisons of detection intensity, they should not be interpreted as molar or mass concentrations. Nevertheless, the profile offers valuable insights into the chemical diversity and relative abundance of phenolic families present in the extract [45,46,47,48].

Among the identified compounds, flavonols were the most prevalent group, with kaempferol showing the strongest presence (100%), followed by quercitrin (65.76%) and isorhamnetin (14.40%). The presence of these compounds in the red corn chaff extract aligns with its tissue origin, which includes crushed leaves, cuticles, and cob fragments. Flavonols are known to play a protective role against UV radiation and oxidative stress in light-exposed tissues [45].

From a functional perspective, the coexistence of kaempferol, quercitrin, isorhamnetin, and their glycosylated derivatives in the extract is particularly noteworthy. These compounds have been individually linked to antioxidant, anti-inflammatory, and cardioprotective activities [45,46,47,48]. However, the true value of the extract lies not just in the properties of each isolated compound, but also in its complex and diverse composition. The potential synergistic or additive effects among flavonols, anthocyanins, and other phenolics could enhance their functionality as natural ingredients. Overall, the phytochemical profile of red corn chaff extract, an underutilized agro-industrial byproduct, suggests a significant potential as a source of bioactive compounds for applications in food or nutraceutical products.

The presence of isorhamnetin (3′-*O*-methylquercetin) and its glycosylated derivatives, such as isorhamnetin-3-*O*-galactoside and isorhamnetin-3-*O*-rhamnoside, further increases the chemical diversity of the extract. The coexistence of these compounds may contribute to additive or synergistic antioxidant and anti-inflammatory effects [49].

Chrysoeriol (3′-*O*-methylluteolin) was identified as one of the most abundant flavonoids, accounting for 35.40% of the relative abundance. This methoxylated derivative of luteolin is commonly found in phenolic-rich vegetables such as basil, celery, and chili peppers, and is particularly prevalent in pigmented maize and other members of the *Poaceae* family [37]. Chrysoeriol has been reported to exhibit antioxidant and antiproliferative activities, as well as protective effects against oxidative DNA damage [50,51].

In addition, eriodictyol, a flavanone, and its glycosylated derivative, eriodictyol-7-*O*-glucoside, were detected, together accounting for 15.54% of the extract’s relative abundance. Flavanones have been associated with anti-inflammatory and hepatoprotective properties, and their presence further enhances the extract’s functional diversity [52].

The extract exhibited substantial structural diversity in its anthocyanin composition, particularly among cyanidin derivatives, including cyanidin-3-(sinapoyl)-glucoside-5-glucoside, cyanidin-3-(6-malonylglucoside)-7-(6-caffeoylglucoside)-3′-glucoside, and cyanidin-3-(3″,6″-dimalonylglucoside). Derivatives of pelargonidin, such as pelargonidin-3-*O*-3″,6″-*O*-dimalonylglucoside, and malvidin, such as malvidin-3-glucoside-5-(6‴-malonyl-2‴-sulfatoglucoside), were also identified. Although not included in Table 1, which lists the 25 most abundant compounds, petunidin and delphinidin derivatives were likewise detected, albeit at lower abundances.

Relative abundance analysis showed that cyanidins and their derivatives accounted for 20.63% of the total extract abundance, whereas all identified anthocyanins collectively represented 30.19%. These results confirm that anthocyanins constitute a substantial fraction of the extract. Nevertheless, the total phenolic content was higher due to the presence of other non-anthocyanin flavonoids, including kaempferol, quercitrin, chrysoeriol, eriodictyol, and various phenolic acids, which collectively exhibited greater signal intensities.

The presence of highly acylated anthocyanins, containing malonyl, sinapoyl, caffeoyl, and sulfate groups, is a distinctive feature of pigmented maize and has important implications for both color expression and extract stability. Cyanidin-derived anthocyanins, which contain two hydroxyl groups on the B ring, produce more intense red and blue hues due to greater electron delocalization than pelargonidin derivatives, which possess a single hydroxyl group and are typically associated with bright red or orange coloration [53].

From a technological perspective, anthocyanin acylation confers enhanced stability through intramolecular copigmentation. In this mechanism, the acyl group forms π-π stacking interactions with the flavylium nucleus, shielding it from nucleophilic attack by water and thereby reducing the rate of colorless chalcone formation [54]. This phenomenon is particularly relevant for food applications, as non-acylated anthocyanins are highly susceptible to degradation induced by changes in pH, temperature, and light exposure. Therefore, the predominance of acylated cyanidin derivatives in the red maize chaff extract suggests that this material may represent a promising source of stabilized natural pigments with intense blue-violet coloration.

This pigmentation pattern is consistent with that reported previously, indicating that cyanidin-derived anthocyanins predominate in red maize, whereas pelargonidin derivatives are the major pigments in pink maize [53]. The detection of multiple mono- and diacylated anthocyanins in the red maize chaff extract not only explains the observed color intensity and stability but also supports its potential application as a functional natural colorant in food matrices with varying acidity and subjected to thermal processing.

### 2.3. Interaction of Anthocyanins with Zein in Liquid Medium

**Figure 2:**
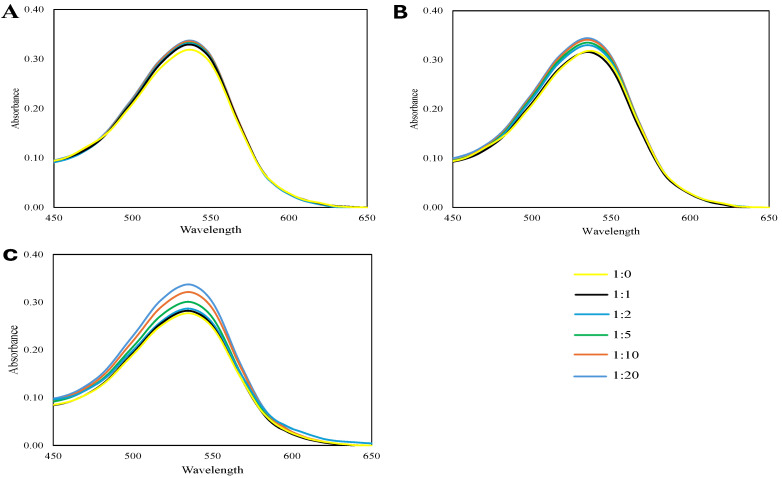
Visible absorption spectra (400–700 nm) of red corn chaff anthocyanins mixed with zein at different anthocyanin:zein ratios (1:0, 1:1, 1:5, 1:10, and 1:20) measured at (**A**) 5 °C, (**B**) 20 °C, and (**C**) 35 °C, pH 3.

Figure 2 presents the visible absorption spectra (λ = 400–700 nm) of anthocyanin extracts obtained from red maize chaff and mixed with zein at different anthocyanin-to-zein ratios (1:0, 1:1, 1:5, 1:10, and 1:20). The spectra were recorded at three temperatures (5, 20, and 35 °C) and at pH 3. Under all conditions, a maximum absorption peak centered at 520 nm was observed, which is characteristic of anthocyanins in their flavylium cation form, the predominant species under acidic conditions [55].

At 5 °C (Figure 2A), increasing the zein proportion (up to an anthocyanin-to-zein ratio of 1:20) resulted in a progressive increase in absorbance at 520 nm, while the position of the absorption maximum remained unchanged. This behavior suggests the formation of anthocyanin–zein copigmentation complexes via noncovalent interactions, such as hydrophobic interactions and hydrogen bonding, without altering the chromophore’s structure [56]. This hyperchromic effect indicates that zein acts as an effective copigment, enhancing color intensity without affecting the wavelength of maximum absorption.

At 20 °C (Figure 2B) and 35 °C (Figure 2C), a similar trend was observed, although the increase in absorbance was less pronounced at higher temperatures, particularly at the 1:20 anthocyanin-to-zein ratio. This behavior can be attributed to two factors: the temperature-dependent interplay between hydrophobic interactions and other non-covalent forces. Within the temperature range studied (5–35 °C), hydrophobic interactions are expected to increase with temperature, as the free energy of transferring nonpolar solutes from a nonpolar environment to water reaches its peak around ~110 °C [57], and the potential partial aggregation of zein near its glass transition temperature, which may reduce the availability of binding sites [38]. However, the observed decrease in the hyperchromic effect at higher temperatures suggests that other factors, such as conformational changes in zein or aggregation, may counteract the strengthening of hydrophobic interactions. This limitation affects the accessibility of binding sites and ultimately reduces the overall copigmentation effect. In all cases, the 1:20 ratio yielded the greatest increase in absorbance, suggesting that a higher zein concentration promotes greater saturation of anthocyanin-binding sites.

It is important to note that, at 20 °C, the spectra for the 1:0 and 1:1 anthocyanin-to-zein ratios are nearly identical. This phenomenon can be explained by the low concentration of zein in the 1:1 ratio (approximately 0.0125 mg mL^−1^), resulting in minimal occupancy of the binding sites. Additionally, at this temperature, hydrophobic interactions are weakened, and there may be some partial aggregation of zein. The difference in absorbance between the two spectra falls within the experimental margin of error (±0.005 absorbance units), indicating that the observed overlap is consistent with the system’s inherent variability and does not affect the interpretation of the results.

No significant bathochromic (towards longer wavelengths) or hypsochromic (towards shorter wavelengths) shifts were observed, indicating that zein does not alter the fundamental electronic structure of anthocyanins. Instead, it exerts an exclusively hyperchromatic stabilizing effect. This behavior is typical of intermolecular copigmentation with proteins, whereas copigmentation with flavonoids often produces bathochromic shifts [28].

The Hill model effectively described the interaction at all temperatures (R^2^ > 0.97). The slopes represent the Hill coefficient (*n*), while the intercepts were used to calculate the binding constant (k) (Table 2).

The results are summarized in Table 2. The binding constant (k) increased with temperature, reaching values of 0.011, 0.017, and 0.033 (mg mL^−1^)^−1^ at 277.15, 293.15, and 318.15 K, respectively. This trend suggests that the association between zein and anthocyanins is thermodynamically favored at higher temperatures. However, this observation appears to contradict the behavior observed in the absorption spectra (Figure 2), where the hyperchromic effect was less pronounced at 35 °C. This apparent discrepancy can be explained by recognizing that the binding constant (k) indicates the intrinsic affinity of the interaction at equilibrium. In contrast, absorption spectra taken at a fixed temperature are affected by the equilibrium concentration of the anthocyanin–zein complex and the molar absorptivity of the species present. The observed decrease in absorbance at higher temperatures, despite an increase in the binding constant, may be attributed to possible aggregation or partial precipitation of zein. This phenomenon reduces the effective concentration of zein available for complex formation and may also alter the local microenvironment of the anthocyanins, thereby affecting their molar absorptivity [38]. Furthermore, temperature-induced conformational changes in zein could alter the binding stoichiometry and the optical properties of the formed complexes.

**Figure 3:**
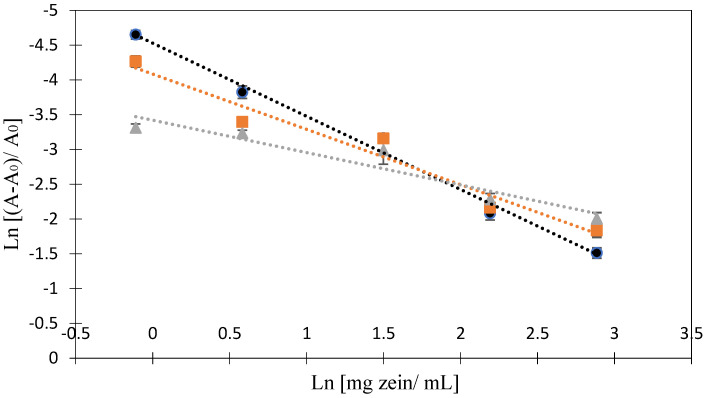
Hill plot for the interaction between zein and anthocyanins from red corn chaff at different temperatures. The graph shows Ln[(A − A_0_)/A_0_] as a function of Ln[zein] (mg mL^−1^) at 277.15 K (●), 293.15 K (■), and 318.15 K (▲). Data points represent the mean ± standard deviation (SD) of three independent measurements. The slopes correspond to the Hill coefficient (*n*), and the intercepts were used to calculate the binding constant (k). Lines represent linear regressions for each temperature (R^2^ > 0.97 in all cases).

Furthermore, the number of binding sites (*n*) progressively decreased with increasing temperature: 1.05 (277.15 K), 0.79 (293.15 K), and 0.47 (318.15 K) (see Figure 3). This trend indicates that at lower temperatures, zein exhibits greater binding capacity, likely due to a more exposed, flexible conformation that enables multiple interactions with anthocyanins. At higher temperatures, zein may undergo conformational changes that hinder access to its hydrophobic sites and hydrogen-bonding networks, thereby decreasing the number of anthocyanins bound per protein molecule [58]. The *n* values close to 1 suggest that, on average, each zein molecule binds to one or two anthocyanin molecules, which is consistent with a simple, non-cooperative, or weakly cooperative stoichiometric interaction.

It is important to note that the Hill coefficient (*n*) at 318.15 K was measured to be 0.465, which is less than 1. While the Hill model is typically used to describe systems with multiple binding sites, in this study, it was used as a phenomenological approximation to characterize the interaction between zein and anthocyanins. The value of *n* < 1 should not be interpreted as indicating a fractional number of binding sites per zein molecule; instead, it suggests that the system deviates from ideal single-site binding behavior.

This deviation can be attributed to several factors: (i) temperature-induced conformational changes in zein that reduce the accessibility of its binding sites [38]; (ii) partial aggregation of zein near its glass transition temperature, which decreases the effective availability of those sites; (iii) heterogeneity within the zein population, as it includes different subunits (α, β, γ, and δ) that have distinct affinities for anthocyanins [37]; and (iv) negative cooperativity effects, where binding of one anthocyanin reduces the affinity of the remaining sites.

Together, these factors explain the progressive decrease in *n* with increasing temperature, dropping from 1.05 at 277.15 K to 0.465 at 318.15 K. Therefore, the *n* value should be understood as an empirical parameter that reflects the effective stoichiometry and degree of cooperativity of the interaction under specific experimental conditions, rather than as an absolute count of binding sites.

The thermodynamic parameters were calculated using the Van’t Hoff equation from the equilibrium constants measured at various temperatures. The results are summarized in Table 2. The Gibbs free energy (ΔG) values were negative at all analyzed temperatures: −3.63 kJ mol^−1^ at 277.15 K, −4.92 kJ mol^−1^ at 293.15 K, and −7.08 kJ mol^−1^ at 318.15 K. These negative ΔG values indicate that the interaction between zein and anthocyanin is thermodynamically spontaneous across the entire temperature range studied [57]. Furthermore, as the temperature increases, ΔG becomes more negative, suggesting that the binding process is favored at higher temperatures, consistent with the observed increase in the equilibrium constant (K).

The entropy change (ΔS) was positive, with a value of 84.5 J mol^−1^ K^−1^. A positive and relatively high entropy contribution is generally associated with the release of structured water molecules from the interaction interface during complex formation, a phenomenon commonly observed in systems where hydrophobic interactions play an important role [59]. In the present study, the association between hydrophobic regions of zein and the aromatic rings of anthocyanins may promote the displacement of water molecules from the protein hydration layer, thereby increasing the number of accessible molecular arrangements in the system and contributing favorably to the entropy gain observed upon binding [59]. The combination of positive ΔH and ΔS values suggests that the interaction is primarily driven by entropic contributions, consistent with hydrophobic interactions, although other non-covalent forces may also contribute to complex formation.

The combination of positive ΔH and ΔS values indicates that the zein–anthocyanin interaction is predominantly driven by favorable entropic contributions, which compensate for the unfavorable enthalpic contribution associated with the endothermic binding process. This entropy gain contributes favorably to the Gibbs free energy term, and positive ΔH and ΔS values have previously been associated with interaction processes dominated by hydrophobic forces [59]. Furthermore, the positive entropy value (ΔS = 84.5 J mol^−1^ K^−1^) suggests that complex formation is thermodynamically favored by solvent reorganization, particularly through the release of structured water molecules from the interaction interface.

These findings are consistent with previous reports on anthocyanin interactions with proteins and copigments. Positive enthalpy (ΔH = 18.45 kJ mol^−1^) and entropy (ΔS = 149.72 J mol^−1^ K^−1^) values, together with negative Gibbs free energy values, have been reported for the interaction between anthocyanins from *Aronia melanocarpa* and bovine serum albumin (BSA), indicating that complex formation was spontaneous and primarily driven by hydrophobic interactions [60]. Similar thermodynamic behavior has been observed during anthocyanin copigmentation processes. For example, a ΔG value of −3.06 kJ mol^−1^ was reported for the interaction between blackberry anthocyanins and ferulic acid, indicating a spontaneous copigmentation process, whereas vanillin exhibited a positive ΔG value of 2.00 kJ mol^−1^, suggesting a thermodynamically less favorable and weaker interaction with anthocyanins [61].

The results suggest that zein interacts with anthocyanins in red corn chaff via a mixed mechanism, driven primarily by hydrophobic interactions (indicated by ΔH > 0 and ΔS > 0), with contributions from hydrogen bonds and van der Waals forces. This interaction pattern has already been described in the context of the relationship between plant proteins and flavonoids [62].

Higher temperatures increased both the binding constant and the spontaneity of the interaction, while reducing the Hill coefficient, suggesting a temperature-dependent balance between binding affinity and the interaction pattern between zein and anthocyanins [41].

From a technological perspective, zein’s ability to co-pigment and stabilize anthocyanins at pH 3, a value commonly encountered in beverages and food products, highlights its potential for developing more stable natural colorants. The favorable thermodynamic parameters obtained in this study, together with the observed interaction between zein and anthocyanins, support the use of zein as a promising stabilizing agent for anthocyanin-based systems. Future studies should focus on elucidating the molecular mechanisms underlying the zein–anthocyanin interaction and evaluating the formation of colloidal aggregates and their impact on color stability during long-term storage. In addition, the performance of these complexes should be assessed in real food matrices and under gastrointestinal conditions to determine their effects on anthocyanin stability and bioaccessibility.

### 2.4. Effect of pH on the Anthocyanin/Zein Interaction

**Figure 4:**
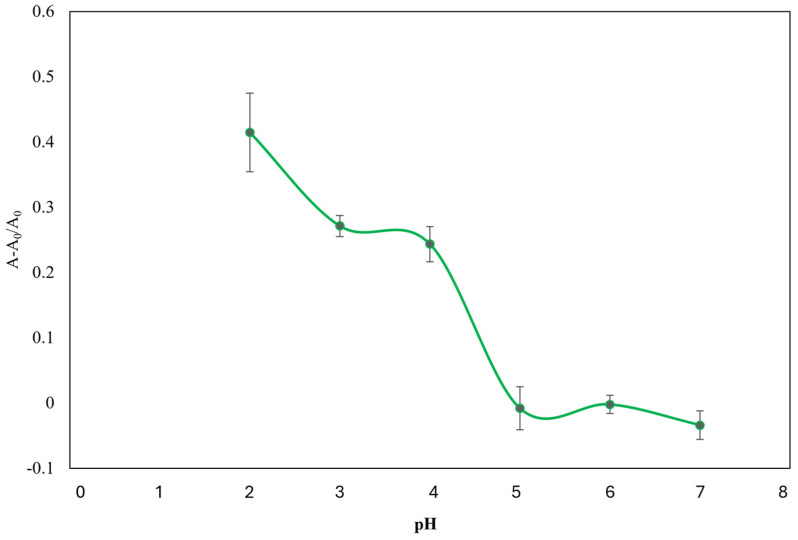
Effect of pH on the copigmentation between anthocyanins extracted from red corn chaff and zein, expressed as the relative increase in absorbance ((A − A_0_)/A_0_) at 520 nm. Measurements were performed at 20 °C (293.15 K) using an anthocyanin-to-zein ratio of 1:20. Data are presented as the mean ± standard deviation (SD) of three independent determinations.

Figure 4 shows how the (A − A_0_)/A_0_ ratio varies with pH, where A_0_ represents the absorbance of the extract without zein and A the absorbance of the extract in the presence of zein (with an anthocyanin-zein ratio of 1:20). Both measurements were performed at 520 nm and 20 °C (293.15 K). This ratio indicates the degree of copigmentation, or the relative increase in absorbance due to the presence of the protein.

The copigmentation effect is strongly dependent on pH. The most significant increase in absorbance is observed under strongly acidic conditions (pH 2), with an (A − A_0_)/A_0_ value close to 0.70. As pH increases, the copigmentation effect gradually decreases; at pH 4, it is reduced by almost half, while at pH 6 and 7 it approaches zero or even becomes slightly negative. This indicates a possible loss of absorbance in the presence of zein, under both neutral and slightly acidic conditions.

With respect to anthocyanin equilibria, at pH 2, these compounds are present predominantly in the flavylium cation form (AH^+^). This species is intensely colored, exhibiting red to red hues, and is generally the most stable form under acidic conditions. Its planar aromatic structure favors interactions with copigments through π-π stacking and hydrogen bonding, thereby promoting copigmentation phenomena [63]. As the pH increases to approximately 4, the equilibrium progressively shifts toward the formation of quinoidal bases, while a fraction of the flavylium cation remains present. Quinoidal bases retain color, typically displaying red to bluish tones, although their relative abundance depends on the pH and anthocyanin structure. At pH 6 and 7, quinoidal base species become increasingly dominant, together with the formation of pseudobase carbinol and chalcone forms. The latter species are weakly colored or colorless and generally less effective in copigmentation interactions, contributing to decreased color intensity and stability observed at near-neutral pH values [54].

Zein, a prolaminin derived from maize, has an estimated isoelectric point around pH 6.2 [37]. At pH 2, zein carries a positive charge due to the protonation of its amino groups. This positive charge increases its solubility and its electrostatic interactions with anthocyanins, which can carry partial positive or negative charges depending on the pH. Furthermore, at acidic pH, zein adopts a more extended conformation, exposing its hydrophobic regions and increasing the accessibility of anthocyanin-binding sites [64].

As the pH approaches its isoelectric point (around pH 6), zein loses its net charge, causing it to aggregate and precipitate. This aggregation significantly reduces its ability to interact with anthocyanins. At pH 7, although zein acquires a net negative charge, its solubility remains limited, and the protein conformation may be less favorable for binding.

The maximum copigmentation effect at pH 2 results from a synergistic combination of interactions: π-π stacking between the aromatic ring of the anthocyanin (in its flavylium cation form) and the aromatic amino acid rings (phenylalanine, tyrosine, tryptophan) of zein, hydrogen bonds between the hydroxyl groups of the anthocyanins and the carbonyl or amide groups of zein, and electrostatic interactions between the positively charged zein and the sulfate or malonyl groups of the acylated anthocyanins (which have a partial negative charge). These interactions further stabilize the complex.

As the pH increases above 4, the concentration of the flavylium form decreases, and the zein begins to lose its positive charge, progressively reducing all these interactions until the copigmentation effect is negligible at neutral pH. Similar findings have been reported on the interaction between anthocyanins and other proteins present in foods. For example, β-lactoglobulin exhibited the greatest hyperchromic effect on grape anthocyanins at pH 3.0, whereas a significant decrease was observed at pH 5.0, and no effect was detected at pH 7.0 [65].

Similar observations have been reported for other protein–anthocyanin systems. Casein has been shown to protect black rice anthocyanins against thermal degradation only at pH values below 4, whereas no protective effect was observed under neutral pH conditions [66]. Regarding zein, previous studies have demonstrated that its ability to form complexes with polyphenols, such as catechins and gallic acid, is greatest at pH 2–3 and becomes nearly negligible at pH 6–7 [32].

The findings suggest that zein is an effective copigment for anthocyanins in acidic food matrices at pH 4 or lower, exhibiting the most pronounced effects in the 2–3 pH range. Although the copigmentation effect decreases by about half at a pH of 4 (Figure 4), the value of (A − A_0_)/A_0_ is approximately 0.35, indicating a considerable increase in absorbance. This improvement can lead to enhanced color stability and intensity in practical applications.

This pH range is relevant for a variety of food products, including fruit juices (pH 3.0–4.0), fruit yogurts (pH 3.8–4.2), gelatins (pH 3.0–4.0), fermented beverages, and certain acidic dairy products. In contrast, in products with a pH closer to neutral, such as milk, meat products, and some baked goods, zein may not aid in color stabilization and could even result in a slight loss of absorbance. This is likely due to the adsorption or precipitation of pigments.

However, its strong copigmenting effect at pH 2–3 suggests that zein could be a promising additive to produce stabilized natural colorants for acidic foods. This offers a plant-based alternative to whey protein or gum arabic, with the added advantage of being a byproduct of the corn industry.

### 2.5. Stability of Anthocyanins in Liquid Medium

The thermal stability of anthocyanins extracted from red corn cob, with and without zein, was evaluated at different temperatures: 4, 20, 35, 50, and 70 °C (equivalent to 277.15, 293.15, 308.15, 323.15, and 343.15 K), and at different pH values (2, 4, 6, and 7) during eight weeks of storage. The results indicate that the zein–anthocyanin complexes exhibited significantly greater stability than the extract without zein across all evaluated conditions. This highlights the protective role of plant protein zein.

**Figure 5:**
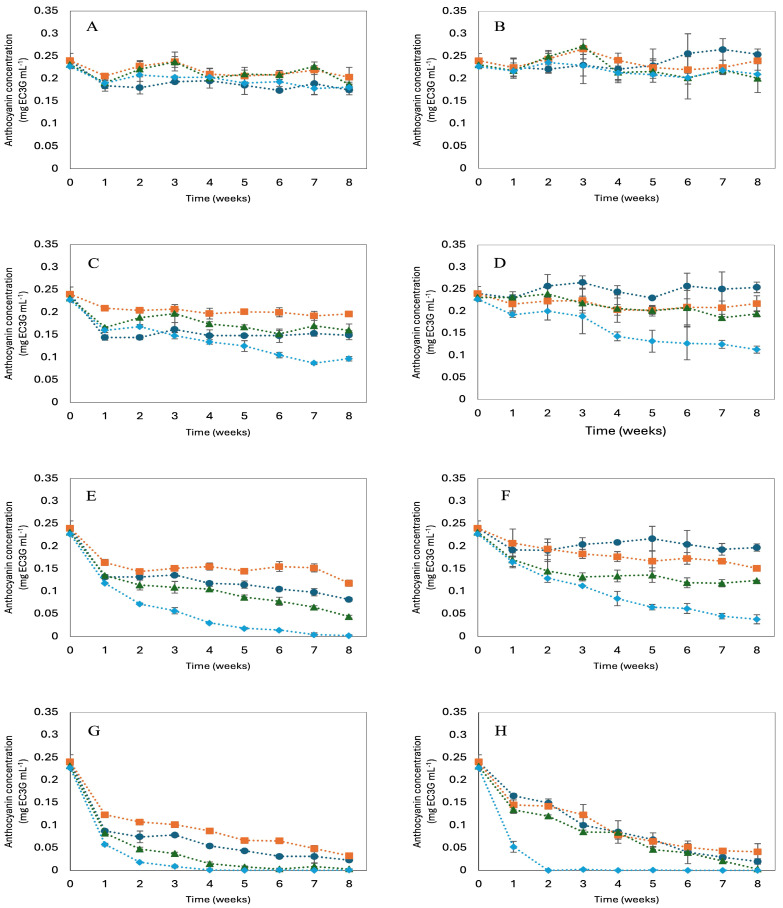
Anthocyanin concentration during storage under different temperature and pH conditions, with and without zein in liquid media. ● pH 2, ■ pH 4, ▲ pH 6, ♦ pH 7. (**A**). 5 °C with zein. (**B**). 5 °C without zein. (**C**). 20 °C with zein. (**D**). 20 °C without zein. (**E**). 35 °C with zein. (**F**). 35 °C without zein. (**G**). 50 °C with zein. (**H**). 50 °C without zein. Values are presented as the mean ± standard deviation (SD) based on three independent measurements.

Figure 5 shows that, at 4 °C, the presence of zein maintains relatively stable anthocyanin concentrations at pH 2 for 8 weeks. This finding suggests that zein provides significant protection to anthocyanins under acidic and low-temperature conditions, where these compounds exist primarily in their stable flavylium cation form [54].

In contrast, at pH 6 and 7, although a slight decrease in anthocyanin concentration was observed, it was considerably smaller than that detected in the extracts without zein. These results indicate that zein can interact with anthocyanins even under near-neutral pH conditions, where these compounds tend to undergo structural transformations toward quinoidal and chalcone forms, which are less stable and more susceptible to degradation [63]. Furthermore, the extracts without zein stored at 4 °C showed a more pronounced decrease in anthocyanin concentration across all pH levels, reinforcing the conclusion that zein exerts a protective effect even at low temperatures.

At 20 °C, the protective effect of zein became more evident. Anthocyanin extracts without zein underwent a substantial reduction in anthocyanin concentration after 8 weeks of storage at all pH levels evaluated, particularly at pH 6 and 7, where degradation occurred more rapidly. In contrast, extracts containing zein also exhibited a gradual decline in anthocyanin concentration, but at a significantly slower rate than those without zein. These results indicate that zein effectively delays anthocyanin degradation, likely through the formation of non-covalent complexes that enhance anthocyanin stability and reduce their susceptibility to degradation reactions [23].

At 35 °C, zein exhibited its most pronounced protective effect at pH 2, 4, and 6. Notably, at pH 4, anthocyanin stability in the liquid system was greater than that observed at pH 2. This finding is particularly relevant from a technological perspective, as pH 4 is common in a variety of food products, including fruit juices, yogurt, and gelatin-based desserts. These results suggest that combining zein with moderately acidic conditions may be a suitable strategy for improving the stability of anthocyanin-based natural colorants.

The greater stability observed at pH 4 in the presence of zein may be attributed to a favorable balance between the physicochemical properties of both components. At this pH, zein remains positively charged, as the medium is below its isoelectric point (pI: 6.2), allowing electrostatic interactions to occur. In addition, the anthocyanin equilibrium at pH 4 includes substantial fractions of both flavylium cations and quinoidal base species, which may promote hydrophobic interactions and π-π stacking with zein more effectively than under the highly acidic conditions found at pH 2 [32].

Finally, at 50 °C, the extracts with and without zein experienced a rapid decrease in anthocyanin concentration over time, although the values were slightly higher in the samples with zein. At this temperature, the thermal energy is sufficient to overcome the energy barriers of noncovalent interactions, leading to complex dissociation and subsequent pigment degradation. However, the fact that the zein-containing samples maintained slightly higher concentrations suggests that, even at elevated temperatures, some residual protection persists, possibly due to steric hindrance or the formation of hydrophobic microenvironments that slow hydrolysis.

It is important to note that within the temperature range of this study (4–50 °C), hydrophobic interactions are expected to strengthen with increasing temperature. However, the protective effect of zein is more pronounced at lower temperatures (4 and 20 °C). This is likely because, at higher temperatures, thermal energy can overcome the energy barriers of non-covalent interactions. As a result, this leads to complex dissociation and subsequent pigment degradation, despite the strengthening of hydrophobic interactions.

The thermoprotective effect of zein on anthocyanins has been attributed to several complementary mechanisms reported in the literature. First, carboxyl-containing amino acid residues, particularly glutamic and aspartic acid residues, may interact with the hydroxyl groups of anthocyanins via hydrogen bonding, thereby stabilizing the colored forms of these pigments. Second, hydrophobic interactions between the aromatic rings of anthocyanins and nonpolar amino acid residues present in zein, such as phenylalanine, leucine, isoleucine, and valine, may promote the formation of non-covalent complexes that reduce the accessibility of water molecules to the anthocyanin chromophore. Such interactions may hinder hydration reactions associated with anthocyanin degradation and color loss [63]. Finally, zein may contribute to anthocyanin stabilization by acting as a physical barrier that limits oxygen diffusion toward the pigment molecules, thereby reducing oxidative degradation [26].

It is important to note that anthocyanins are particularly unstable at near-neutral pH due to their tendency to tautomerize between quinoidal and chalcone forms. These compounds are susceptible to degradation by thermal rupture, oxidation, or hydrolysis [62]. However, the results of this study show that zein can form stable interactions with anthocyanins even at pH 6 and 7, improving overall stability compared to protein-free extracts. This finding is significant, as most studies on protein copigmentation have focused on acidic pH, and there is limited information on zein–anthocyanin interactions at neutral pH [32].

Table 3 presents a comparative analysis of the degradation rate constants (k, h^−1^), coefficients of determination (R^2^), half-life values (t_1/2_, h), and activation energies (E_a_, kJ mol^−1^) obtained for anthocyanin extracts with and without zein under different pH and temperature conditions. In all cases, R^2^ values exceeded 0.70 and were close to or greater than 0.90 in most treatments. These results indicate that the degradation data were adequately described by a first-order kinetic model, in agreement with previous studies on anthocyanins from various plant sources [67,68].

At pH 2 and 277.15 K (4 °C), the extract without zein exhibited a degradation rate constant (k) of 9 × 10^−5^ h^−1^ and a half-life (t_1/2_) of 7702 h (approximately 321 days), indicating a high degree of stability even in the absence of protein. In the presence of zein, however, the degradation rate constant decreased to 1 × 10^−5^ h^−1^, while the half-life increased to 69,315 h (approximately 7.9 years). This result corresponds to an approximately nine-fold increase in half-life, demonstrating a substantial improvement in anthocyanin stability. As the temperature increased to 293.15, 308.15, and 323.15 K, k values progressively increased in both systems, reflecting an acceleration of thermal degradation. Nevertheless, across all evaluated temperature conditions, samples containing zein consistently exhibited lower k values and higher t_1/2_ values than those without zein, demonstrating the protein’s persistent protective effect at pH 2.

At pH 4 and 277.15 K (4 °C), the extracts without zein exhibited a degradation rate constant (k) of 4 × 10^−5^ h^−1^ and a half-life (t_1/2_) of 17,329 h (approximately 722 days). Under the same conditions, the presence of zein resulted in a similar k value (4 × 10^−5^ h^−1^), indicating that the protective effect was not evident at this temperature. However, significant differences emerged at higher temperatures. For example, at 308.15 K (35 °C), the extract without zein exhibited a k value of 5 × 10^−4^ h^−1^ and a half-life of 1386 h, whereas the zein-containing extract showed a lower k value of 3 × 10^−4^ h^−1^ and a longer half-life of 2311 h, corresponding to an increase in half-life of approximately 67%. This finding is particularly relevant from a technological perspective, as pH 4 is commonly encountered in beverages and fermented food products, while 35 °C represents a typical storage temperature in warm climates. The enhanced stability observed at pH 4 in the presence of zein suggests that moderately acidic conditions may be particularly favorable for stabilizing anthocyanins in food systems.

At pH 6, clear differences were observed between extracts with and without zein. At 277.15 K, the extract without zein exhibited a degradation rate constant (k) of 6 × 10^−5^ h^−1^ and a half-life (t_1/2_) of 11,552 h, whereas the zein-containing extract showed a slightly higher k value of 1 × 10^−4^ h^−1^. However, at elevated temperatures (308.15 and 323.15 K), samples containing zein exhibited substantially lower k values than the corresponding protein-free extracts. For example, at 308.15 K (35 °C), the extract without zein showed a k value of 1.0 × 10^−3^ h^−1^ and a half-life of 693 h, whereas the zein-containing extract exhibited a k value of 4 × 10^−4^ h^−1^ and a half-life of 1733 h, corresponding to a 2.5-fold increase in half-life. This finding is particularly relevant because pH 6 represents a near-neutral condition under which free anthocyanins are generally unstable due to equilibrium shifts toward quinoidal base, pseudobase carbinol, and chalcone species, which are more susceptible to degradation [63]. The ability of zein to enhance anthocyanin stability even at pH 6 suggests that non-covalent interactions remain effective under these conditions and is consistent with the contribution of hydrophobic and π-π interactions to complex formation and stabilization.

At pH 7, free anthocyanins are highly unstable, particularly at elevated temperatures. At 323.15 K (50 °C), the degradation rate constant (k) is 7.2 × 10^−3^ h^−1^, and the half-life (t_1/2_) is only 96 h (4 days). However, in the presence of zein, the degradation rate constant decreases to 6.2 × 10^−3^ h^−1^, and the half-life increases to 112 h (4.7 days). Although this improvement is modest in absolute terms, it represents a 16% increase in half-life under extremely unfavorable conditions. This finding suggests that even at neutral pH, zein can form stable interactions that slow down the degradation of anthocyanins, possibly through steric hindrance that impedes the nucleophilic attack of water on the pyrylium ring [58].

The activation energy (E_a_), calculated using the Arrhenius equation, indicates the sensitivity of anthocyanin degradation rates to temperature changes. As shown in Table 3, extracts without zein exhibited higher E_a_ values than those containing zein at all pH conditions evaluated. These results indicate that the temperature dependence of anthocyanin degradation was generally greater in the absence of zein, whereas zein reduced the degradation process’s sensitivity to temperature variations.

A higher E_a_ value indicates that the degradation rate is more strongly temperature-dependent, suggesting that the system is more susceptible to thermal acceleration. In contrast, the lower E_a_ values observed for the zein-containing samples indicate a reduced thermal sensitivity of the degradation process. These results suggest that interactions between zein and anthocyanins modify the degradation pathway, thereby reducing the temperature dependence of the degradation rate. Consequently, anthocyanin degradation in the presence of zein is less affected by temperature fluctuations than in protein-free systems.

For example, at pH 2, the activation energy (E_a_) decreased from 57.21 kJ mol^−1^ in the absence of zein to 35.6 kJ mol^−1^ in its presence. Similarly, E_a_ decreased from 59.36 to 39.17 kJ mol^−1^ at pH 4 and from 64.44 to 43.88 kJ mol^−1^ at pH 6, corresponding to a reduction of approximately 26%. This latter decrease is particularly noteworthy, as it coincided with a 2.5-fold increase in half-life at pH 6 and 308.15 K in the zein-containing samples compared with the protein-free extracts. The E_a_ values obtained in this study are within the range previously reported for blackcurrant anthocyanins (60–85 kJ mol^−1^) and *Hibiscus sabdariffa* anthocyanins (approximately 70–90 kJ mol^−1^) [67,68].

Overall, the results indicate that the presence of zein modifies the temperature dependence of anthocyanin degradation and reduces the degradation process’s sensitivity to temperature fluctuations during storage. Together with the lower degradation rate constants and longer half-lives observed in the zein-containing systems, these findings support the role of zein as an effective stabilizing agent for anthocyanins.

As shown in Table 3, zein, a low-cost and widely available byproduct of the corn industry, can be used as a natural stabilizer for anthocyanins in liquid media, especially at pH 4 and when stored at refrigeration (4 °C) or room temperature (20 °C). The longer half-life (t_1/2_) and lower activation energy (E_a_) in the presence of zein highlight its potential to extend the shelf life of natural pigments in food applications. This characteristic could lead to the development of more stable natural food colorants, thereby reducing reliance on synthetic additives or expensive microencapsulation systems.

### 2.6. Evolution of Antioxidant Capacity During Storage

**Figure 6:**
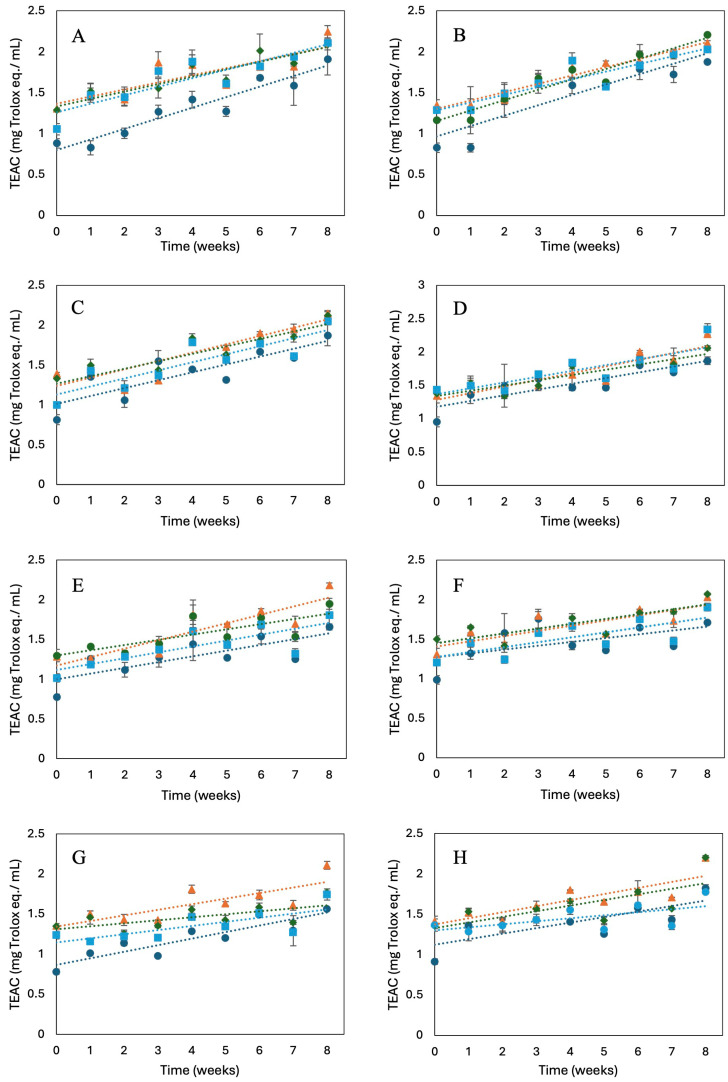
Trolox equivalent capacity (mg Trolox mL^−1^) behavior during storage. ■ pH 2, ● pH 4, ♦ pH 6, ▲ pH 7. (**A**) 5 °C with zein. (**B**) 5 °C without zein. (**C**) 20 °C with zein. (**D**) 20 °C without zein. (**E**) 35 °C with zein. (**F**) 35 °C without zein. (**G**) 50 °C with zein. (**H**) 50 °C without zein. Values are presented as the mean ± standard deviation (SD) based on three independent measurements.

Figure 6 illustrates the evolution of antioxidant capacity, expressed as milligrams of Trolox equivalents per milliliter of extract (mg Trolox mL^−1^), during 8 weeks of storage under different temperature (5, 20, 35, and 50 °C) and pH (2, 4, 6, and 7) conditions, in the presence and absence of zein. Figure 6A,C,E,G correspond to extracts containing zein, whereas Figure 6B,D,F,H correspond to extracts without zein at the respective temperatures.

Interestingly, antioxidant capacity remained stable or even increased over time across all evaluated temperature and pH conditions, despite the progressive decline in anthocyanin concentration observed during storage (Figure 5). The increase in antioxidant capacity was particularly pronounced at pH 6 and 7, conditions under which anthocyanin degradation was most extensive in the liquid system. At 5 °C (Figure 6A,B), both zein-containing and protein-free samples exhibited nearly constant antioxidant capacity throughout the 8-week storage period, regardless of pH, with no significant differences observed between treatments. At 20 °C (Figure 6C,D), a slight increase in antioxidant capacity became evident after the fourth week of storage, particularly at pH 6 and 7. This increase was more pronounced in the samples without zein (Figure 6D) than in those containing zein (Figure 6C). These results suggest that the presence of zein may slow anthocyanin degradation, as shown in Figure 5, thereby reducing the rate of formation of degradation products that retain or contribute to antioxidant activity.

At 35 °C (Figure 6E,F), the differences became more evident: in the samples without zein (Figure 6F), antioxidant capacity increased progressively from the second week, reaching peak levels between weeks 6 and 8, especially at pH 6 and 7 (with increases of up to 2.5 times the initial value). In contrast, the samples with zein (Figure 6E) showed a more moderate increase, reaching a maximum of approximately 1.5 times the initial value.

At 50 °C (Figure 6G,H), the most dynamic changes were documented. In the samples without zein (Figure 6H), antioxidant capacity increased rapidly during the first 2 to 4 weeks, particularly at pH 6 and 7 (up to 3.5-fold), then stabilized or slightly decreased around the eighth week, possibly due to degradation of secondary phenolic compounds. In the samples containing zein (Figure 6G), the increase was more gradual and less pronounced, reaching a maximum of approximately 2 times the initial value, indicating greater stability at the achieved levels.

The effect of pH on antioxidant capacity was evident throughout the storage period. At pH 2 and 4, antioxidant capacity remained relatively stable across all temperatures evaluated, with only slight increases observed at 50 °C toward the end of the storage period. This behavior is consistent with the greater stability of anthocyanins under acidic conditions, which limits their degradation during storage. In contrast, the most pronounced increases in antioxidant capacity were observed at pH 6 and 7, particularly at elevated temperatures (35 and 50 °C) and in the absence of zein. Notably, these conditions coincided with the highest anthocyanin degradation rates reported in Table 3. For example, at pH 6 and 323.15 K, the degradation rate constant reached 0.0039 h^−1^ in the absence of zein, with a half-life of 178 h, whereas at pH 7 the corresponding values were 0.0072 h^−1^ and 96 h, respectively. The association between increased antioxidant capacity and enhanced anthocyanin degradation suggests that degradation products may contribute to the overall antioxidant activity of the extracts.

Several mechanisms may explain this apparently paradoxical behavior. First, the hydrolytic or thermal degradation of anthocyanins can release acylating groups from their structures, including phenolic acids such as sinapic, caffeic, and ferulic acids, as well as malonic acid (Table 1). The released phenolic acids exhibit well-documented antioxidant activity and may help maintain or even enhance the extracts’ overall antioxidant capacity during storage.

A second possible mechanism involves the copigmentation of the remaining anthocyanins with phenolic acids released during degradation. These compounds may form non-covalent complexes through π-π stacking and hydrogen-bonding interactions, thereby stabilizing anthocyanins and providing additional antioxidant activity. As a result, synergistic effects between anthocyanins and phenolic acids may contribute to the antioxidant capacity of the extracts [15,63].

From a molecular recognition perspective, the interaction between anthocyanins and phenolic acids is governed by structural and electronic complementarity between both molecules. Anthocyanins, in their flavylium or quinoidal base forms, possess a delocalized π system that acts as an electron acceptor, while phenolic acids (such as caffeic, ferulic, or sinapic acid) have aromatic rings with hydroxyl and methoxyl groups that donate electron density. This complementarity favors π-π stacking and the formation of hydrogen bonds between the hydroxyl groups of phenolic acids and the carbonyl or hydroxyl groups of anthocyanins, resulting in supramolecular complexes that are more stable than individual anthocyanin aggregates [53].

Previous studies have demonstrated that copigmentation with phenolic acids not only improves the thermal and photochemical stability of anthocyanins but also modulates their antioxidant activity. For example, Sendri et al. [69] demonstrated that copigmentation of cyanidin-3-*O*-glucoside with chlorogenic acid and caffeic acid significantly increases the antioxidant capacity of the system, an effect attributed to the formation of supramolecular complexes that facilitate electron and hydrogen atom transfer. Similarly, it has been reported that the presence of phenolic acids can modify the tautomeric equilibrium of anthocyanins toward more stable forms with higher antioxidant activity [15].

Furthermore, Zhao et al. [70] investigated the interaction between black rice anthocyanins and phenolic acids using computational and experimental approaches, demonstrating that binding affinity depends on both the phenolic acid’s structure (number and position of hydroxyl groups) and the medium’s pH. Their findings revealed that phenolic acids with more hydroxyl and methoxyl groups exhibit higher binding constants and more pronounced copigmentation effects, which correlate with greater protection against thermal degradation. These results are consistent with the composition of the red corn chaff extract analyzed in the present study, in which sinapic, caffeic, and ferulic acids were identified (Table 1), which could exert a copigmentation effect like that observed in the systems.

In the context of our study, the release of phenolic acids during the thermal degradation of acylated anthocyanins could generate an “auto-copigmentation” effect, in which the degradation products themselves act as copigments for the remaining anthocyanins. This phenomenon would explain why the antioxidant capacity of the extract is maintained or even increases despite the decrease in anthocyanin concentration (Figure 6). Furthermore, the presence of zein could modulate this process by selectively stabilizing certain molecular species or by creating a microenvironment that favors copigmentation interactions [32]. These findings highlight the importance of molecular recognition in stabilization and suggest that the design of anthocyanin-based antioxidant systems should consider not only pigment concentration but also the composition of endogenous and exogenous copigments, which can enhance their functionality.

Another contributing factor may be the formation of degradation intermediates, including chalcones and phenolic aldehyde derivatives, some of which have been reported to retain or even exhibit higher antioxidant activity than the parent anthocyanins. In this regard, Nayak et al. [71] reported that thermally treated sweet potato anthocyanins exhibited a significant increase in the Trolox equivalent antioxidant capacity (TEAC)-to-anthocyanin ratio, indicating that degradation products contributed substantially to the measured antioxidant capacity.

Finally, a fourth possible mechanism involves the protective effect exerted by zein. Previous studies have suggested that zein may stabilize anthocyanins through hydrophobic interactions and hydrogen bonding [72]. This protection may slow anthocyanin degradation and modulate the increase in antioxidant capacity observed during storage. Purified sweet potato anthocyanins subjected to thermal treatments above 100 °C have been reported to maintain their antioxidant capacity and, in some cases, to exhibit an increase when expressed as the TEAC-to-anthocyanin ratio. This increase was attributed to the formation of thermal degradation intermediates with enhanced antioxidant activity [71]. Similarly, yeast-derived protein has been shown to improve anthocyanin stability, resulting in comparable antioxidant capacity values before and after thermal treatment. Furthermore, this protein protected anthocyanins during heating at 80 °C, providing additional evidence that protein–anthocyanin interactions can enhance anthocyanin stability [73].

The discrepancy between anthocyanin concentration and antioxidant capacity has important methodological and technological implications. On the one hand, while anthocyanin concentration (measured by differential pH) is a direct indicator of the pigment’s structural integrity, it does not necessarily reflect the extract’s antioxidant functionality. On the other hand, while anthocyanins can degrade during storage (especially at pH 6–7 and elevated temperatures), the extract may maintain or even increase its antioxidant capacity. This suggests that such materials could continue to function as functional ingredients (sources of natural antioxidants) even after significant pigment loss, particularly in applications where color is not a critical attribute.

### 2.7. Color Stability

Color changes in concentrated red corn chaff extracts were evaluated under different storage temperatures (4, 20, 35, and 50 °C) and pH conditions (2, 4, 6, and 7), in both the presence and absence of zein. The results are summarized in Table 4, which presents the CIELAB color coordinates (L*, a*, and b*), and in Figure 6, which shows the total color difference (ΔE) during storage.

The untreated extract exhibited pH-dependent color characteristics. At pH 2, the CIELAB coordinates were L* = 20.50, a* = 35.50, and b* = 3.68, indicating the intense red-purple coloration typically associated with the flavylium cation. At pH 4, the values were a* = 29.00 and b* = −2.52, corresponding to violet hues. At pH 6, a* decreased to 27.42, while b* decreased to −5.56, resulting in a more bluish coloration. At pH 7, the coordinates were a* = 17.28 and b* = −11.35, producing a pale blue-violet color consistent with the predominance of quinoidal base species and the increased contribution of hydrated and chalcone forms.

At 4 °C, extracts lacking zein maintained their initial color values, whereas the addition of zein caused significant changes (*p* < 0.05). For instance, at pH 7, the a* coordinate, which indicates red color intensity, increased from 18.66 to 32.73, while the b* coordinate, which represents blue color, decreased from −8.33 to 1.33. These changes suggest a notable enhancement in red coloration and a reduction in blue tones, indicating that zein modified the color expression of the anthocyanin system through interactions between the protein and anthocyanins.

Chemically, these effects can be explained by several concurrent molecular mechanisms. Firstly, at pH 7, anthocyanins mainly exist as quinoidal bases, which retain a planar aromatic structure. Zein, a protein rich in hydrophobic amino acids (such as leucine, alanine, proline, and phenylalanine), has nonpolar regions that can interact with the delocalized π system of quinoidal bases via π-π stacking. These interactions stabilize the colored form of anthocyanins, shifting the equilibrium toward quinoidal species and decreasing the formation of colorless forms (such as the carbinol pseudobase and chalcone) [63,73].

Moreover, the hydrophobic environment created by zein around anthocyanins lowers the local dielectric constant, discouraging hydration of the pyrylium ring—the reaction responsible for producing carbinol pseudobase—and stabilizing charged or partially charged species by reducing solvation [54,71].

Additionally, the phenolic hydroxyl groups of quinoidal bases can form hydrogen bonds with the carbonyl groups (–C=O) in the zein peptide chain, as well as with the hydroxyl groups of serine and threonine residues and the amino groups (–NH_2_) of lysine residues. While these hydrogen bonds are weak individually, they act cooperatively to enhance the stability of the protein–anthocyanin complex [32,62].

Despite pH 7 being close to the isoelectric point of zein (pI ≈ 6.2), local charges remain on the protein surface due to the uneven distribution of acidic and basic residues. These charges can interact electrostatically with the sulfate and malonyl groups of the acylated anthocyanins found in this extract, further stabilizing the complex [54].

Finally, zein’s binding to anthocyanins creates steric hindrance that restricts water molecules’ access to the C-2 carbon of the pyrylium ring, a critical site for the hydration reaction that leads to the formation of the colorless carbinol pseudobase. This physical protection significantly reduces the degradation rate [23,58].

In summary, these mechanisms explain why zein can provide a moderate but significant stabilizing effect on anthocyanins, even at pH 7 (a condition that is typically unfavorable for free anthocyanins). This effect is reflected in an increase in the a* coordinate (indicating a stronger red hue) and a decrease in the b* coordinate (indicating a weaker blue hue), suggesting a shift toward more stable and colorful forms of anthocyanins.

On the other hand, at 20 °C, samples without zein exhibited reductions in both L* and a* values. For example, at pH 6, a* decreased from 27.42 to 25.00, likely because of anthocyanin degradation (Table 4). In contrast, the zein-containing samples exhibited higher lightness and red coloration. At pH 6, L* increased from 15.67 to 21.29, and a* increased from 25.00 to 29.60, while b* values remained relatively stable (from −10.31 to −3.33). These results suggest that zein helps preserve color characteristics and may reduce color deterioration associated with storage and temperature exposure [22,72].

At 35 °C, the increases in a* and b* values were more pronounced in the samples containing zein. For example, at pH 2, the color coordinates reached L* = 24.66, a* = 44.45, and b* = 15.51, indicating a brighter red-orange coloration than that observed in the control samples. A similar trend was observed at pH 4 (a* = 34.32, b* = 14.33) and pH 6 (a* = 28.71, b* = 5.66). These color changes are consistent with enhanced intermolecular interactions between zein and anthocyanins and may reflect copigmentation under moderate heating conditions.

The most pronounced color changes were observed at 50 °C. Although anthocyanin concentrations decreased substantially under these conditions (Table 4; for example, at pH 2 and 323.15 K, k = 0.0018 h^−1^ and t_1/2_ = 385 h in the presence of zein), the zein-containing samples exhibited the highest a* value recorded in the entire study, reaching 62.66 at pH 2. These samples also displayed L* and b* values of 18.43 and 14.00, respectively. This apparent contradiction may be explained by several complementary mechanisms. First, the formation of zein–anthocyanin complexes may help preserve color expression through non-covalent interactions [30].

Second, the release of phenolic acids from acylated anthocyanins during thermal degradation may promote copigmentation with the remaining anthocyanins, thereby enhancing color intensity [32]. Third, thermal processing may favor the formation of more stable-colored derivatives, including polymeric pigments such as pyranoanthocyanin-like compounds, which have been associated with enhanced color stability and intensity [74]. Finally, interactions between zein and anthocyanins may alter the local microenvironment surrounding the pigments, potentially shifting the equilibrium toward more intensely colored anthocyanin species [41].

**Figure 7:**
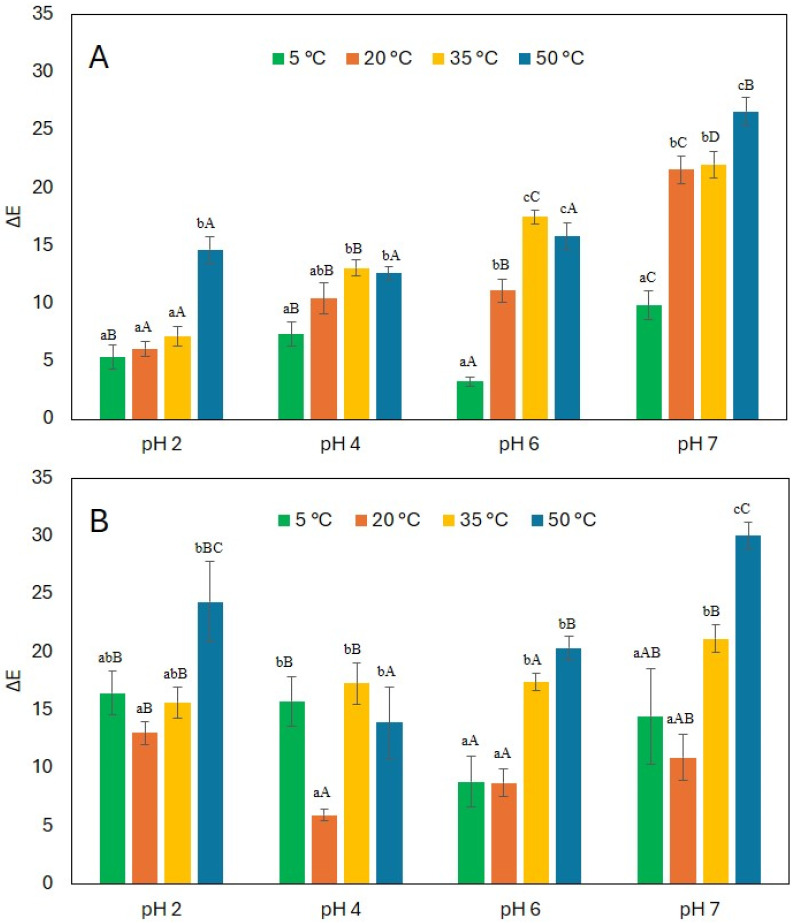
Total color change of the extracts (ΔE) at different pH levels after heat treatment under different pH levels, (**A**) without zein and (**B**) with zein. Different lowercase letters indicate significant differences between temperatures within the same pH (*p* < 0.05). Different capital letters indicate significant differences between pH within the same temperature (*p* < 0.05). Values are presented as the mean ± standard deviation (SD) based on three independent measurements.

Figure 7 shows the total color difference (ΔE), which quantifies the visual perception of color changes relative to the untreated extract used as the reference sample, under different pH (2, 4, 6, and 7) and temperature (4, 20, 35, and 50 °C) conditions for extracts with and without zein. In the samples without zein (Figure 7A), a gradual increase in ΔE was observed, with both pH and temperature influencing the magnitude of the color change. Notably, the greatest overall color differences were observed at pH 6 and 7, whereas significantly smaller changes were detected at pH 2 and 4. This behavior is consistent with the lower stability of anthocyanins under near-neutral conditions. At pH 6 and 7, equilibrium shifts toward quinoidal base, hydrated, and chalcone species have been associated with a progressive loss of the original color characteristics of anthocyanin extracts, resulting in larger overall color differences [54,63].

Temperature also had a pronounced effect on ΔE at all pH levels. The highest ΔE values were observed in samples exposed simultaneously to elevated pH and temperature, highlighting the combined influence of these factors on anthocyanin degradation and associated changes in color properties during storage [67].

The addition of zein to the extracts (Figure 7B) produced noticeable changes in overall color variation, particularly in samples stored at low temperature (4 °C). In these instances, the systems containing zein exhibited higher ΔE values than the corresponding protein-free extracts. It is essential to understand that ΔE measures the extent of perceived color change relative to a reference point but does not indicate whether this change signifies deterioration (loss of color) or enhancement (intensification or modification of color). Since ΔE was calculated based on the untreated control for each system, these differences suggest that zein influenced the color evolution of the anthocyanin extracts during storage. At low temperatures, the increase in ΔE observed with the addition of zein does not indicate pigment degradation. Instead, it reflects a positive change in color coordinates, consistent with the increases in a* (red hue) and b* (yellow hue) shown in Table 4. This indicates a copigmentation effect that enhances and modifies the color towards more vibrant red-orange tones. This behavior is likely related to interactions between proteins and anthocyanins, including hydrophobic interactions and hydrogen bonding, which can alter the system’s optical response and improve its color stability over time [72]. This effect was observed across all pH levels but was most pronounced at acidic pH (2 and 4), where the largest increases in ΔE were observed.

During these conditions, zein displays its maximum copigmenting effect. Conversely, at higher temperatures (35 and 50 °C), the ΔE values in zein-containing systems were more moderate than in the protein-free extracts, particularly at pH 4. Under these conditions, the presence of zein moderates the increase in ΔE observed during storage, indicating a stabilizing effect on the system’s color properties. This dual behavior of zein, characterized by an initial modification of color characteristics followed by a reduction in the magnitude of thermally induced color changes, suggests that protein–anthocyanin interactions influence the system’s response to environmental conditions.

It is important to note that a high ΔE value does not necessarily indicate pigment degradation; variations in ΔE should be interpreted as changes in overall color perception. In the presence of zein, the increase in ΔE observed at low temperatures was associated with intensified red coloration, as evidenced by the higher positive a* and b* values reported in Table 4. At elevated temperatures, the color changes observed in zein-containing systems may reflect not only anthocyanin degradation but also the formation of new molecular associations among zein, residual anthocyanins, and phenolic acids released during thermal degradation [15,71].

These results demonstrate that zein significantly affects the color properties of the extracts, helping mitigate color changes across different environmental conditions. Zein exhibited protective effects at intermediate pH (4) and low storage temperatures (4–20 °C), reducing the extent of color degradation during storage. At higher temperatures (35–50 °C), although greater color changes were observed, these changes remained controlled and were associated with the development of red-orange hues that may be technologically desirable. Therefore, zein may serve as an effective color-modulating and chroma-stabilizing agent in anthocyanin-containing systems. Its behavior is influenced by both pH and temperature, making it a promising low-cost, plant-derived alternative for applications involving thermally processed foods.

## 3. Materials and Methods

The Mexican red corn landrace chaff used in this study was donated by engineer Everardo Lovera Gómez, the general director of the Agricultural, Aquaculture and Forestry Research and Training Institute (ICAMEX) in the Atlacomulco region of the State of Mexico. The material obtained as a by-product of corn threshing was presented in small flakes. The chaff was stored in a dry place, at room temperature (22 ± 2 °C), protected from light. The zein used in this study was purchased from Sigma-Aldrich Chemical Co. (St. Louis, MO, USA), with CAS number 9010-66-6. Similarly, analytical reagents such as DPPH, Trolox, and the Folin–Ciocalteu reagent were purchased from Sigma-Aldrich Chemical Co. (St. Louis, MO, USA). The rest of the reagents were of analytical grade.

### 3.1. Optimization of the Extraction

To maximize the recovery of phenolic compounds, a Box–Behnken experimental design with 3 factors and three levels each was used: temperature (20, 40, and 60 °C), ethanol concentration (20, 50, and 80% *w*/*v*), and solid: solvent ratio (1:1, 10:1 and 20:1). The response variables were the concentration of phenolic compounds and anthocyanins and the antioxidant capacity. Optimal extraction conditions were determined by maximizing only the anthocyanin concentration using Design-Expert software (version 13) (Stat-Ease, Inc., Minneapolis, MN, USA).

### 3.2. Effect of Zein Concentration on Protein/Anthocyanin Interaction

The optimized extract, based on anthocyanin concentration, was used to evaluate the effect of different anthocyanin:zein ratios (1:0, 1:1, 1:5, 1:10, and 1:20) on absorbance at 520 nm. Absorbance measurements were performed using a Multiskan GO microplate spectrophotometer (Thermo Fisher Scientific, Vantaa, Finland). The mixtures were agitated using an UltraRocker rocking platform (Bio-Rad Laboratories, Hercules, CA, USA) at 1000 rpm, 20 °C for 24 h, protected from light. Based on the data obtained, the following were calculated: the Gibbs free energy (∆G), the association constant (k), the number of binding sites (*n*), and the stoichiometric relationship of the association between zein and anthocyanin, using the Hill equation [61]:

Ln[(A − A_0_)/A_0_] = Ln(k) + *n* Ln(C)

where A_0_ is the absorbance of the extract in the absence of zein (λ = 520 nm), A is the absorbance of the extract in the presence of zein (λ = 520 nm), k is the apparent equilibrium constant of the zein/anthocyanin interaction process, *n* represents the anthocyanin-to-zein stoichiometric ratio (Hill constant), and C is the zein concentration in mg mL^−1^.

ΔG = −RT Ln(K)

where T is the absolute temperature (K), and R is the universal gas constant (8.314 J mol^−1^ K^−1^). The enthalpy change (ΔH) associated with the interaction between anthocyanins and the protein was determined using the Van’t Hoff equation.

d(LnK)/d(1/T) = −(ΔH/R)

All thermodynamic parameters are presented as the mean ± standard error (SE) based on three independent measurements. The errors for the parameters k and *n* were derived from the standard errors obtained from the linear regression of the Hill plot. The errors for ΔG were calculated using error propagation based on the uncertainties in K. Additionally, the errors for ΔH and ΔS were determined from the standard errors of the slope and intercept of the Van’t Hoff plot.

### 3.3. Effect of pH on Protein/Anthocyanin Interaction

The effect of pH on copigmentation between zein and anthocyanins was evaluated using an anthocyanin-to-zein ratio of 1:10 (*w*/*v*), and the absorbance was measured at 520 nm (Multiskan GO microplate spectrophotometer, Thermo Fisher Scientific, Vantaa, Finland). The results were expressed as the color difference (ΔE) between samples with and without zein in the CIE Lab color space. The pH of the medium was adjusted using 0.1 N HCl or NaOH solutions.

### 3.4. Degradation Kinetics of the Phenolic Compounds in the Extract

The optimized anthocyanin extract was used for the thermal treatment study. Four extracts without zein were prepared, and their pH values were adjusted to 2, 4, 6, and 7 using 1 N HCl or 1 N NaOH solutions. Another set of extracts was prepared at the same pH values by adding zein at a 1:10 ratio relative to the total phenolic concentration.

Samples were placed in sealed containers and stored at different temperatures to evaluate the effects of temperature on the concentrations of phenolic compounds and anthocyanins. Extracts stored at 4 °C were kept in a temperature-controlled refrigerator (4 °C). Samples stored at room temperature (22 ± 2 °C) were protected from light throughout the storage period. The remaining extracts were placed in a temperature-controlled oven set at 35, 50, or 70 °C.

During eight consecutive weeks, with weekly measurements, the following parameters were determined in each extract (with and without zein): total phenolic content, total anthocyanin content, in vitro antioxidant capacity, and color coordinates. The degradation data for phenolic compounds and anthocyanins were fitted to a first-order kinetic model to obtain the degradation rate constant (k, h^−1^) and half-life (t_1/2_) values using the following equations:

Ln(C_t_/C_0_) = −kt

t_1/2_ = Ln 2/k

where Ct is the anthocyanin concentration at storage time t (h), and C0 is the anthocyanin concentration in the fresh extract. The activation energy (E_a_, J mol^−1^) and the pre-exponential factor (A, h^−1^) were calculated using the linearized Arrhenius equation:

Ln(k) = Ln(A) − (E_a_/R) (1/T)

where R is the universal gas constant (J mol^−1^ K^−1^), and T is the storage temperature (K) [7].

The degradation rate constants (k) and the half-life values (t_1/2_) are presented as the mean ± standard error (SE), which were calculated from fitting the first-order kinetic model to the experimental data (n = 3). The activation energies (E_a_) were determined from the slope of the Arrhenius plot (ln k versus 1/T), and the associated errors were calculated based on the standard error of the linear regression. The coefficients of determination (R^2^) reflect the goodness of fit for each kinetic model.

### 3.5. Phytochemical Analysis of the Extract by FIA-ESI-FTICR-MS

Mass spectra of the compounds present in the extracts were acquired using flow injection analysis–electrospray ionization coupled with Fourier transform ion cyclotron resonance mass spectrometry (FIA-ESI-FTICR-MS) on a Solarix XR 7T instrument (Bruker, Bremen, Germany). Analyses were performed in both positive and negative ionization modes. Samples were diluted 1:10 in an ethanol–water mixture and injected at a flow rate of 120 μL/h. Sodium trifluoroacetate was used for mass calibration. Spectra were acquired over an *m*/*z* range of 50–2000, with 100 scans collected at an accumulation time of 0.3 s per scan. Nitrogen was used as the nebulizing gas at a pressure of 0.6 bar, a flow rate of 4 L min^−1^, and a temperature of 180 °C. Data were processed using DataAnalysis v6.0 and MetaboScape 2022b software. Compound annotation was performed using the ChemSpider and PubChem databases [75].

The mass error (ppm) was calculated as: Error (ppm) = [(*m*/*z*_experimental − *m*/*z*_theoretical)/*m*/*z*_theoretical] × 10^6^. Compound identification was performed by comparing experimental m/z values with those of compounds listed in the ChemSpider and PubChem databases, using a mass tolerance of ≤5 ppm. The relative abundance of each identified compound was determined by normalizing its intensity to that of the compound with the highest intensity in the extract, which was set to 100%.

### 3.6. Quantification of Total Phenolic Compounds

Total phenolic content was determined using the Folin–Ciocalteu method adapted to a 96-well microplate format, as described by Singleton et al. [76]. Briefly, 20 μL of the sample was mixed with 100 μL of Folin–Ciocalteu reagent diluted 1:5 with water. After incubation at room temperature for 5 min, 80 μL of 10% (*w*/*v*) Na_2_CO_3_ solution was added. The mixture was incubated for 30 min to allow color development. Absorbance was measured at 765 nm, and the results were expressed as milligrams of gallic acid equivalents per gram of dry sample (mg GAE g^−1^) using a calibration curve prepared with gallic acid as the standard.

### 3.7. Quantification of Total Anthocyanins

Total anthocyanin content was determined using the pH differential method described by Giusti and Wrolstad [54], adapted for microplate-based measurement. Briefly, 20 μL of diluted extract (1:10) was mixed with 180 μL of sodium acetate buffer (CH_3_COONa, pH 4.5) in one well. In a separate well, the same volume of diluted sample was mixed with 180 μL of KCl buffer at pH 1.0. Both mixtures were incubated at room temperature for 5 min, after which absorbance was measured at 510 nm (the maximum absorption wavelength of cyanidin-3-glucoside) and at 700 nm. The results were expressed as milligrams of cyanidin-3-glucoside equivalents (C3G) per gram of dry weight (mg C3G g^−1^ DW) according to the following equation:

Total anthocyanin content (mg C3G g^−1^ sample) = (A × MW × DF × V)/ε × L × W

where A = [(A510 − A700)_pH1.0_ − (A510 − A700)_pH4.5_], MW is the molecular weight of C3G (449.2 g mol^−1^), DF is the dilution factor, V is the volume of the obtained extract (mL), ε is the molar absorptivity of cyanidin-3-*O*-glucoside (26,900 L mol^−1^ cm^−1^), L is the path length (cm), and W is the amount of dry sample used for extraction (g).

### 3.8. In Vitro Antioxidant Activity (DPPH Assay)

Antioxidant capacity was evaluated using the DPPH free radical scavenging assay described by Brand-Williams et al. [77], adapted to a 96-well microplate format. A 0.152 mM DPPH solution was prepared by dissolving 1.5 mg of DPPH in 25 mL of methanol. In each well, 10 μL of sample, standard, or blank was mixed with 190 μL of the DPPH solution. The mixture was incubated at room temperature in the dark for 30 min. Subsequently, absorbance was measured at 517 nm. The results were expressed as milligrams of Trolox equivalents per milliliter (mg TE mL^−1^) using a calibration curve prepared with Trolox (0–0.3 mg mL^−1^).

### 3.9. Color Measurement of the Extracts

Color coordinates in the CIELab color space were determined using a Color Reader C-10 tristimulus colorimeter (Konica Minolta Optics Inc., Chiyoda, Tokyo, Japan). The L* (lightness), a* (positive values indicate red hues and negative values indicate green hues), and b* (positive values indicate yellow hues and negative values indicate blue hues) coordinates were recorded. In addition, the total color difference (ΔE) was calculated using the initial L*_0_, a*_0_, and b*_0_ coordinates of each extract at time 0 for each storage condition as reference values, according to the following equation:

ΔE = √[(L* − L_0_)^2^ + (*a* − a_0_)^2^
*+* (*b* − b*_0_)^2^]

### 3.10. Statistical Analysis

All chemical analyses were performed at least in triplicate, and the results are presented as the mean ± standard deviation (SD). Differences among treatments were evaluated by analysis of variance (ANOVA), and differences were considered significant at *p* < 0.05. All statistical analyses were performed using Minitab Statistical Software version 22.1.0 (Minitab, LLC, State College, PA, USA).

## 4. Conclusions

The interaction between zein and anthocyanins extracted from red maize cobs was found to be thermodynamically spontaneous (ΔG < 0), endothermic (ΔH > 0), and predominantly driven by hydrophobic interactions (ΔS > 0). This interaction promotes the formation of stable complexes under acidic conditions (pH 2–4). Zein was observed to decrease the degradation rate constant of anthocyanins and increase their half-life, particularly at pH 4 and storage temperatures of 4 °C and 20 °C, demonstrating a pronounced protective effect on these phenolic compounds.

The lower activation energy (E_a_) of the zein–anthocyanin complex, compared with that of the extract in the absence of zein, indicates reduced thermal sensitivity of the stabilized system. In addition, zein enhances red coloration, as evidenced by increased a* values, and positively influences the overall color difference (ΔE). At the same time, it preserves and even increases the extract’s antioxidant capacity—by up to 3.5-fold—under accelerated degradation conditions. This improvement may be associated with the release of phenolic acids by exhibiting radical-scavenging activity.

The optimized extract obtained from red maize chaff, rich in cyanidin-derived acylated anthocyanins, represents a promising source of natural pigments. These findings indicate that zein, a low-cost byproduct of the maize industry, can effectively act as a heat- and color-stabilizing agent for anthocyanins in acidic food matrices with a pH of 4 or lower. Its stabilizing effect is particularly strong within the pH range of 2 to 3, although it still shows a notable copigmenting effect at pH 4 (~0.35, (A − A_0_)/A_0_). This pH range includes many commercially important food products, such as fruit juices, yogurts, gelatins, and fermented beverages. Consequently, zein offers a plant-based alternative to animal- or synthetic-based stabilizers for creating more stable natural colorants. To confirm its potential for industrial use, further research on the molecular interactions between zein and anthocyanins, along with evaluations in real food matrices, is recommended. Further structural studies on zein–anthocyanin interactions, as well as evaluations of their performance in real food systems, are recommended to confirm their industrial applicability.

## Figures and Tables

**Table 1:** Phenolic compounds identified in red corn chaff extracts by FIA-ESI-FTICR-MS, with relative abundances normalized to the baseline peak *.

Compound Name	Bucket Label	*m*/*z*	Intensity	Expected [M + H]^+^	Error [ppm]	Relative Abundance [%]
Kaempferol-3-*O*-(6-malonyl-glucoside)	534.10	535.11	124,000,000.00	535.11	−0.29	100.00
Quercitrin	448.10	449.11	81,545,305.00	449.11	0.56	65.76
Chrysoeriol-7-*O*-(6-malonyl-glucoside)	548.12	549.13	43,900,000.00	549.13	−0.76	35.40
Kaempferol-3-*O*-glucuronide	462.12	463.13	34,400,000.00	463.13	−0.46	27.74
Eriodictyol-7-*O*-glucoside	450.12	451.13	19,270,387.00	451.13	−0.26	15.54
Isorhamnetin-3-*O*-galactoside	478.15	479.16	17,854,702.00	479.16	−0.40	14.40
Cyanidin-3-(sinapoyl)-glucoside-5-glucoside	817.20	818.21	9,786,018.00	818.21	0.01	7.89
1-Sinapoyl-2-feruloylgentiobiose	724.19	725.20	8,344,898.90	725.20	0.00	6.73
Dicaffeoylquinic acid	516.07	517.08	8,024,761.50	517.08	0.00	6.47
Kaempferol-3-*O*-acetyl-glucoside	490.12	491.12	6,072,511.30	491.12	−0.32	4.90
Isorhamnetin-3-*O*-rutinoside	462.11	463.12	5,048,775.50	463.12	0.01	4.07
Cyanidin-3-(6-malonylglucoside)-7-(6-caffeoylglucoside)-3′-glucoside	1020.17	1021.18	5,027,588.50	1021.18	0.00	4.05
Epicatechin-(2a-7)(4a-8)-epicatechin 3-*O*-Gal	706.16	707.16	4,912,963.80	707.16	0.01	3.96
Pelargonidin-3-*O*-3″,6″-*O*-dimalonylglucoside	604.07	605.08	3,486,190.50	605.08	0.00	2.81
Isorhamnetin-3-*O*-glucoside-7-*O*-rhamnoside	624.15	625.16	3,335,225.80	625.16	0.00	2.69
Kaempferol	286.03	287.04	3,080,436.10	287.04	0.00	2.48
Quercetin-3-*O*-xylosyl-glucuronide	610.02	611.03	2,940,558.50	611.03	0.01	2.37
Cyanidin-3-(3″,6″-dimalonylglucoside)	620.07	621.07	2,467,979.80	621.07	0.32	1.99
1,2,2-Triferuloylgentiobiose	870.21	871.21	2,431,118.00	871.21	0.00	1.96
3-4-Diferuloylquinic acid	544.10	545.11	2,401,698.20	545.11	0.00	1.94
Malvidin-3-glucoside-5-(6‴-malonyl-2‴-sulfatoglucoside)	820.14	821.14	2,136,027.00	821.14	0.00	1.72
Quercetin-3-sulfate	380.07	381.08	1,978,064.40	381.08	−0.01	1.59
3,4-Dicaffeoyl-1,5-quinolactone	498.12	499.13	1,877,751.50	499.13	0.00	1.51
4-Hydroxybenzoic acid 4-*O*-glucoside	300.09	301.10	1,689,005.50	301.10	0.02	1.36
Cyanidin 3-*O*-beta-D-(P-coumaroyl)-sambubioside	726.18	727.19	1,681,889.40	727.19	0.01	1.36

* Note: The 25 compounds with the highest relative abundance are listed, including flavonols, flavones, flavonones, and acylated anthocyanins. The bucket label represents the mass-to-charge ratio (*m*/*z*) value used as a reference for compound identification. *m*/*z* = refers to the experimental mass-to-charge ratio of the detected ion. Intensity corresponds to the absolute abundance of the ion signal, measured in counts per second (cps). Expected [M + H]^+^ denotes the theoretical m/z value calculated from the molecular formula of the protonated compound. The error, expressed in parts per million (ppm), indicates mass accuracy, calculated as the difference between the experimental and theoretical *m*/*z* values, divided by the theoretical *m*/*z* value, and then multiplied by 10^6^. Relative abundance [%] is determined by normalizing each compound’s intensity to the most intense signal (kaempferol 3-*O*-(6-malonylglucoside), assigned a value of 100%).

**Table 2:** Kinetic and thermodynamic parameters associated with temperature and zein/anthocyanin interactions.

T (°K)	k	*n*	K	ΔG (kJ mol^−1^)	ΔH (kJ mol^−1^)	ΔS (J mol^−1^ K^−1^)
277.15	0.0108 ± 0.0007	1.051 ± 0.025	4.85 ± 0.31	−3.63 ± 0.15	19.8 ± 1.16	84.5 ± 6.6
293.15	0.0168 ± 0.0005	0.795 ± 0.036	7.55 ± 0.22	−4.92 ± 0.07		
318.15	0.0326 ± 0.0012	0.465 ± 0.02	14.65 ± 0.54	−7.08 ± 0.1		

k = Binding constant (apparent equilibrium constant from Hill plot), *n* = Hill coefficient (number of binding sites), K = equilibrium constant (dimensionless), ΔG = Gibbs free energy, ΔH = enthalpy, ΔS = entropy. Values are presented as the mean ± standard deviation (SD) based on three independent measurements. The values of ΔH and ΔS were calculated from the slope and intercept of the Van’t Hoff plot, respectively, with error estimates derived from linear regression.

**Table 3:** Comparative analysis of degradation rate constants (k), coefficients of determination (R^2^), half-life values (t_1/2_), and activation energies (E_a_) of the extract in the absence and presence of zein under varying pH and temperature conditions.

pH	T [K]	Without Zein				with Zein			
		k [h^−1^]	R^2^	t_1/2_ [h]	E_a_ [kJ mol^−1^]	k [h^−1^]	R^2^	t_1/2_ [h]	E_a_ [kJ mol^−1^]
2	277.15 (4 °C)	9 ×10^−5^	0.79	7701.60 ± 286	57.21 ± 3.47	1 × 10^−5^	0.70	69,314.7 ± 1917	35.6 ± 5.91
	293.15 (20 °C)	3 × 10^−4^	0.90	2310.40 ± 182		3 × 10^−5^	0.77	23,104.9 ± 1027	
	308.15 (35 °C)	6 × 10^−4^	0.91	1155.20 ± 141		6 × 10^−5^	1.00	11,552.4 ± 656	
	323.15 (50 °C)	1.5 × 10^−3^	0.98	462.01 ± 39		1.8 × 10^−3^	0.95	385.1 ± 63	
4	277.15 (4 °C)	4 × 10^−5^	0.96	17,328 ± 2278	59.36 ± 7.63	4 × 10^−5^	0.88	17,328.6 ± 956	39.17 ± 1.79
	293.15 (20 °C)	1 × 10^−4^	0.97	6931.40 ± 411		7 × 10^−5^	0.97	9902.1 ± 449	
	308.15 (35 °C)	5 × 10^−4^	0.92	1386.20 ± 252		3 × 10^−4^	0.91	2310.5 ± 293	
	323.15 (50 °C)	1.2 × 10^−3^	0.96	577.60 ± 47		1.3 × 10^−3^	0.96	533.2 ± 72	
6	277.15 (4 °C)	6 × 10^−5^	0.88	11,552.40 ± 874	64.44 ± 10.17	1 × 10^−4^	0.95	6931.4 ± 1600	43.88 ± 10.15
	293.15 (20 °C)	2 × 10^−4^	0.89	3465.70 ± 201		2 × 10^−4^	0.82	3465.7 ± 357	
	308.15 (35 °C)	1 × 10^−3^	0.91	693.10 ± 33		4 × 10^−4^	0.84	1732.8 ± 273	
	323.15 (50 °C)	3.9 × 10^−3^	0.98	177.70 ± 51		1.8 × 10^−3^	0.94	385.1 ± 72	
7	277.15 (4 °C)	1 × 10^−4^	0.88	6931.40 ± 1546	71.15 ± 3.94	7 × 10^−5^	0.75	9902.1 ± 806	55.85 ± 5.47
	293.15 (20 °C)	6 × 10^−4^	0.96	1155.20 ± 65		5 × 10^−4^	0.90	1386.36 ± 158	
	308.15 (35 °C)	3.3 × 10^−3^	0.96	210.10 ± 50		1.3 × 10^−3^	0.99	533.2 ± 35	
	323.15 (50 °C)	7.2 × 10^−3^	0.96	96.20 ± 32		6.2 × 10^−3^	0.90	111.8 ± 31	

The data are expressed as the mean ± standard deviation (SD) from three independent measurements. The R^2^ values represent the coefficient of determination for the fitting of the first-order kinetic model. The E_a_ values were calculated using the Arrhenius plot, and the associated errors were determined through linear regression.

**Table 4:** CIELab color coordinates (L*, a*, and b*) of red corn chaff extracts subjected to thermal treatment (4–50 °C) at different pH values (2–7) with and without zein.

Storage Conditions			L*	a*	b*	Color
Untreated	pH 2		20.50 ± 2.12	35.5 ± 2.12	3.68 ± 4.24	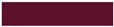
	pH 4		15.50 ± 0.71	29.00 ± 0.00	−2.52 ± 2.12	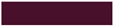
	pH 6		14.17 ± 4.24	27.42 ± 1.41	−5.56 ± 1.41	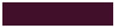
	pH 7		13.37 ± 2.82	17.28 ± 2.28	−11.35 ± 1.41	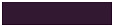
4 °C	pH 2	Without zein	19.66 ± 1.52	39.66 ± 1.27 *	10.33 ± 0.57 *	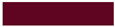
		With zein	18.33 ± 0.48	33.33 ± 0.89	16.00 ± 2.00 *	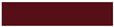
	pH 4	Without zein	21.15 ± 1.06	26.45 ± 1.17 *	−1.33 ± 1.52	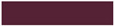
		With zein	13.06 ± 0.05 *	31.65 ± 1.56	14.33 ± 2.08 *	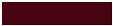
	pH 6	Without zein	12.33 ± 1.52 *	28.33 ± 0.57	−6.33 ± 1.34	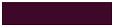
		With zein	12.28 ± 2.42 *	21.66 ± 0.81 *	−2.46 ± 1.15 *	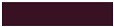
	pH 7	Without zein	6.66 ± 1.15 *	18.66 ± 1.15	−8.33 ± 0.52	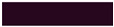
		With zein	13.67 ± 3.21	32.73 ± 0.76 *	1.33 ± 3.50 *	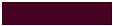
20 °C	pH 2	Without zein	18.56 ± 0.74 *	38.43 ± 1.32 *	6.00 ± 1.00	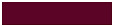
		With zein	20.33 ± 0.61	39.00 ± 1.00 *	18.01 ± 0.90 *	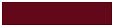
	pH 4	Without zein	11.32 ± 0.63	23.05 ± 2.53	5.00 ± 2.63 *	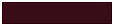
		With zein	21.21 ± 0.58 *	29.05 ± 0.07	−3.00 ± 0.88	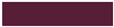
	pH 6	Without zein	15.67 ± 1.52	25.00 ± 1.00 *	−10.31 ± 0.45 *	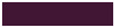
		With zein	21.29 ± 1.81 *	29.60 ± 0.21	−3.33 ± 0.57	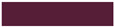
	pH 7	Without zein	16.01 ± 1.00	23.73 ± 0.97 *	3.66 ± 0.49 *	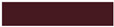
		With zein	13.59 ± 0.45 *	21.21 ± 2.08 *	−2.46 ± 2.08 *	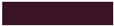
35 °C	pH 2	Without zein	17.30 ± 0.05 *	36.36 ± 0.43	11.05 ± 0.09 *	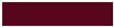
		With zein	24.66 ± 1.52 *	44.45 ± 1.12 *	15.51 ± 0.38 *	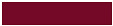
	pH 4	Without zein	11.75 ± 0.39 *	26.34 ± 0.61 *	8.71 ± 0.58 *	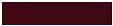
		With zein	15.30 ± 1.49	34.32 ± 2.43 *	14.33 ± 1.52 *	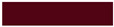
	pH 6	Without zein	15.15 ± 0.57	31.00 ± 1.00 *	5.03 ± 1.11 *	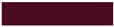
		With zein	20.11 ± 1.60 *	28.71 ± 3.05	5.66 ± 0.57 *	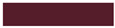
	pH 7	Without zein	11.34 ± 1.52 *	27.01 ± 0.97 *	1.46 ± 0.46 *	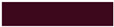
		With zein	16.59 ± 1.31 *	31.02 ± 0.07 *	3.33 ± 1.49 *	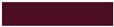
50 °C	pH 2	Without zein	28.33 ± 0.57 *	48.31 ± 1.89 *	15.28 ± 0.61 *	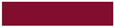
		With zein	18.43 ± 0.68	62.66 ± 3.21 *	14.00 ± 2.00 *	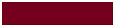
	pH 4	Without zein	18.67 ± 0.41 *	37.37 ± 0.08 *	4.44 ± 0.08 *	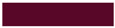
		With zein	17.01 ± 1.00 *	37.33 ± 2.51 *	8.00 ± 2.00 *	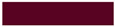
	pH 6	Without zein	14.09 ± 0.06	33.33 ± 1.52 *	4.00 ± 0.00 *	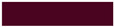
		With zein	16.33 ± 1.62 *	34.33 ± 1.52 *	12.66 ± 2.08 *	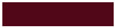
	pH 7	Without zein	13.42 ± 0.65	33.00 ± 1.00 *	9.57 ± 2.01 *	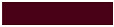
		With zein	19.22 ± 2.08 *	32.32 ± 2.04 *	12.03 ± 0.08 *	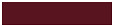

Data are presented as mean ± standard deviation (SD) from three determinations. The asterisk (*) denotes statistically significant differences compared to the untreated extract (control) (*p* < 0.05). The “Color” column provides an approximate visual representation for each condition.

## Data Availability

The data presented in this study are available on request from the corresponding authors.

## References

[1] Ðordevic T., Antov M. (2018). Wheat chaff utilization: Evaluation of antioxidant capacity of waste streams generated by different pretreatments. Ind. Crops Prod..

[2] Bian Q., Ambrose R., Subramanyam B. (2015). Effect of chaff on bulk flow properties of wheat. J. Stored Prod. Res..

[3] Bava A., Carnelli S., Vaccari M., Beffa T., Beltrametti F. (2025). Identification of corn chaff as an optimal substrate for the production of rhamnolipids in *Pseudomonas aeruginosa* fermentations. Fermentation.

[4] Kusuma H., Zahra K., Saputri R., Utomo M., Jaya D., Amenaghawon A., Darmokoesoemo H. (2024). Unlocking the potential of agricultural waste as biochar for sustainable biodiesel production: A comprehensive review. Bioresour. Technol. Rep..

[5] Gallegos D., Wedwitschka H., Moeller L., Zehnsdorf A., Stinner W. (2017). Effect of particle size reduction and ensiling fermentation on biogas formation and silage quality of wheat straw. Bioresour. Technol..

[6] Wang M., Qiao J., Sheng Y., Wei J., Cui H., Li X., Yue G. (2023). Bioconversion of corn fiber to bioethanol: Status and perspectives. Waste Manag..

[7] Weber F., Boch K., Schieber A. (2017). Influence of copigmentation on the stability of spray-dried anthocyanins from blackberry. LWT-Food Sci. Technol..

[8] Fernandez F., Hernandez L., Aguilar G., Arrieta D., Navarro A. (2019). Extraction and identification of anthocyanins in corn cob and corn husk from cacahuacintle maize. J. Food Sci..

[9] Mohammadi N., Franchin M., Girotto C., Greggi L., Granato D. (2024). Green recovery and application of berry anthocyanins in functional gummies: Stability study, plasma and cellular antioxidant and anti-inflammatory activity. Food Res. Int..

[10] Li X., Wang Y., Jiang Y., Liu C., Zhang W., Chen W., Tian L., Sun J., Lai C., Bai W. (2024). Microencapsulation with fructooligosaccharides and whey protein enhances the antioxidant activity of anthocyanins and their ability to modulate gut microbiota in vitro. Food Res. Int..

[11] Escobedo A., Avalos L., Mojica L., Lugo E., Gschaedler A., Alcazar M. (2024). Native Mexican black bean purified anthocyanins fractionated by high-performance counter-current chromatography modulate inflammatory pathways. Food Chem..

[12] Chen T., Xie L., Wang G., Jiao J., Zhao J., Yu Q., Chen Y., Shen M., Wen H., Ou X. (2024). Anthocyanins-natural pigment of colored rice bran: Composition and biological activities. Food Res. Int..

[13] Gradinaru G., Biliaderis C., Kallithraka S., Kefalas P., García C. (2003). Thermal stability of *Hibiscus sabdariffa* L. anthocyanins in solution and in solid state: Effects of copigmentation and glass transition. Food Chem..

[14] Kanha N., Surawang S., Pitchakarn P., Laokuldilok T. (2020). Microencapsulation of copigmented anthocyanins using double emulsion followed by complex coacervation: Preparation, characterization and stability. LWT-Food Sci. Technol..

[15] Wang J., Zhao Y., Sun B., Yang Y., Wang S., Feng Z., Li J. (2024). The structure of anthocyanins and the copigmentation by common micromolecular copigments: A review. Food Res. Int..

[16] Da Cruz Nascimento S., Rodrigues T., dos Santos M., Medeiros R., Florentino K., Fernandes C., Souza T., de Souza F. (2025). Oat-grape beverages enriched with anthocyanins from jambolan (*Syzygium cumini*): A novel plant-based probiotic functional food. Food Chem..

[17] He J., Carvalho A., Mateus N., De Freitas V. (2010). Spectral features and stability of oligomeric pyranoanthocyanin-flavanol pigments isolated from red wines. J. Agric. Food Chem..

[18] Wu J., Guan Y., Zhong Q. (2015). Yeast mannoproteins improve thermal stability of anthocyanins at pH 7.0. Food Chem..

[19] Fan L., Wang Y., Xie P., Zhang L., Li Y., Zhou J. (2019). Copigmentation effects of phenolics on color enhancement and stability of blackberry wine residue anthocyanins: Chromaticity, kinetics and structural simulation. Food Chem..

[20] He Z., Xu M., Zeng M., Qin F., Chen J. (2016). Preheated milk proteins improve the stability of grape skin anthocyanin extracts. Food Chem..

[21] Guan Y., Zhong Q. (2015). The improved thermal stability of anthocyanins at pH 5.0 by gum arabic. LWT-Food Sci. Technol..

[22] Chung C., Rojanasasithara T., Mutilangi W., McClements D. (2017). Stability improvement of natural food colors: Impact of amino acid and peptide addition on anthocyanin stability in model beverages. Food Chem..

[23] Chung C., Rojanasasithara T., Mutilangi W., McClements D. (2015). Enhanced stability of anthocyanin-based color in model beverage systems through whey protein isolate complexation. Food Res. Int..

[24] Feitosa B., Angioletti B., Sousa E., Camargo M., Rodrigues S., Barros L. (2025). Anthocyanins stability theory—Evidence summary on the effects of microencapsulation. Food Bioprod. Process..

[25] Sun J., Zhang L., Huo J., Zhang Y., Sui X. (2025). Enhancing anthocyanin stability in blue honeysuckle berries via interaction with whey protein isolate: Structural and spectroscopic insights. Food Chem..

[26] Li T., Wang L., Chen Z., Zhang X., Zhu Z. (2020). Functional properties and structural changes of rice proteins with anthocyanins complexation. Food Chem..

[27] Sui X., Sun H., Qi B., Zhang M., Li Y., Jiang L. (2018). Functional and conformational changes to soy proteins accompanying anthocyanins: Focus on covalent and non-covalent interactions. Food Chem..

[28] Eyiz V., Tontul I., Türker S. (2026). Production of stable anthocyanin-based food colorants from *Hibiscus sabdariffa* L. by copigmentation with different protein isolates. J. Food Meas. Charact..

[29] Kurek M.A., Pokorski P., Aktaş H., Custodio-Mendoza J.A. (2025). Tailoring anthocyanin stability with pea–rice proteins and fructooligosaccharides: A microencapsulation study using double emulsion. Food Bioprod. Process..

[30] Zhang Z., Tang S., Li Z., Chou S., Shu C., Chen Y., Chen W., Yang S., Yang Y., Tian J. (2022). An updated review on the stability of anthocyanins regarding the interaction with food proteins and polysaccharides. Compr. Rev. Food Sci. Food Saf..

[31] Yang M., Zhang M., Wang B., Zhang Z., Zhuang Y., Liu J., Zhang Q., Fei P. (2025). Mechanism-driven stabilization of anthocyanins: Comparative copigmentation and encapsulation for food applications. Food Chem..

[32] Li S., Wang X., Zhang X., Zhang H., Li S., Zhou J., Fan L. (2023). Interactions between zein and anthocyanins at different pH: Structural characterization, binding mechanism and stability. Food Res. Int..

[33] Tian Q., Tian J., Li Z., Quintana R., Yang B., Wang L., He Y., Li B. (2026). Binding characteristics and mechanisms underlying the enhanced stability of anthocyanins by proteins screened with molecular docking. Food Chem..

[34] Li Z., Tian J., Tian Q., Zang Z., Wang Y., Jiang Q., Chen Y., Yang B., Yang S., Yang Y. (2025). Improved uptake of anthocyanins-loaded nanoparticles based on phenolic acid-grafted zein and lecithin. Food Chem..

[35] Zheng H., Liu G., Wang L., Zhou W., Zhang X., Fu Y. (2026). Co-Encapsulation of *Lonicera caerulea* L. Anthocyanins and Gallic Acid within Zein-Gum Arabic Nanoparticles: Characterization, Stability, In Vitro Digestion, and Antioxidant Activity. Food Biophys..

[36] Yan X., Yan J., Zhu J., Xing F., Gu Q., Gao J., Zhao C., Liu J. (2025). Structural properties and antioxidant activities of zein during in vitro simulated gastrointestinal digestion: Focus on effect of corn post-ripening. LWT-Food Sci. Technol..

[37] Shukla R., Cheryan M. (2001). Zein: The industrial protein from corn. Ind. Crops Prod..

[38] Zhang Y., Wu C., Fang X., Xu L., Shen H., Song M., Zhang H., Luan G. (2025). Structural transitions and molecular interactions of zein induced by ethanol and extrusion. Food Chem. X.

[39] Nour V., Štampar F., Veberič R., Jakopič J. (2013). Anthocyanins profile, total phenolics and antioxidant activity of black currant ethanolic extracts as influenced by genotype and ethanol concentration. Food Chem..

[40] Cacace J.E., Mazza G. (2003). Mass transfer process during extraction of phenolic compounds from milled berries. J. Food Eng..

[41] Enaru B., Drețcanu G., Pop T.D., Stǎnilǎ A., Diaconeasa Z. (2021). Anthocyanins: Factors affecting their stability and degradation. Antioxidants.

[42] Li J., Shi C., Shen D., Han T., Wu W., Lyu L., Li W. (2022). Composition and antioxidant activity of anthocyanins and non-anthocyanin flavonoids in blackberry from different growth stages. Foods.

[43] Petroni K., Pilu R., Tonelli C. (2014). Anthocyanins in corn: A wealth of genes for human health. Planta.

[44] Paulsmeyer M., Chatham L., Becker T., West M., West L., Juvik J. (2017). Survey of anthocyanin composition and concentration in diverse maize germplasms. J. Agric. Food Chem..

[45] Imran M., Rauf A., Shah Z.A., Saeed F., Imran A., Arshad M.U., Ahmad B., Bawazeer S., Atif M., Peters D.G. (2019). Chemo-preventive and therapeutic effect of the dietary flavonoid kaempferol: A comprehensive review. Phytother. Res..

[46] Chen A.Y., Chen Y.C. (2013). A review of the dietary flavonoid, kaempferol on human health and cancer chemoprevention. Food Chem..

[47] Septembre-Malaterre A., Boumendjel A., Seteyen A.-L.S., Boina C., Gasque P., Guiraud P., Sélambarom J. (2022). Focus on the high therapeutic potentials of quercetin and its derivatives. Phytomed. Plus.

[48] Xu D., Hu M.-J., Wang Y.-Q., Cui Y.-L. (2019). Antioxidant activities of quercetin and its complexes for medicinal application. Molecules.

[49] Da Silva A.P., da Silva Cordeiro M.L., de Queiroz Aquino-Martins V.G., Scortecci K.C., Jha A.K., Dubey D.K. (2026). Role of Flavonoids and Their Potential Application in Health. Natural Products: Phytochemistry, Botany, Metabolism of Alkaloids, Phenolics and Terpenes.

[50] Mishra B., Priyadarsini K.I., Kumar M.S., Unnikrishnan M.K., Mohan H. (2003). Effect of O-glycosilation on the antioxidant activity and free radical reactions of a plant flavonoid, chrysoeriol. Bioorg. Med. Chem..

[51] Aboulaghras S., Sahib N., Bakrim S., Benali T., Charfi S., Guaouguaou F.-E., Omari N.E., Gallo M., Montesano D., Zengin G. (2022). Health Benefits and Pharmacological Aspects of Chrysoeriol. Pharmaceuticals.

[52] Yin H., Li Y., Feng Y., Tian L., Li Y. (2024). The Extraction, Biosynthesis, Health-Promoting and Therapeutic Properties of Natural Flavanone Eriodictyol. Nutrients.

[53] Peniche-Pavía H.A., Tiessen A. (2020). Anthocyanin profiling of maize grains using DIESI-MSI QTOF reveals that cyanidin-based derivatives predominate in purple corn, whereas pelargonidin-based molecules occur in red-pink varieties from Mexico. J. Agric. Food Chem..

[54] Giusti M.M., Wrolstad R.E. (2003). Acylated anthocyanins from edible sources and their applications in food systems. Biochem. Eng. J..

[55] Giusti M.M., Wrolstad R.E. (2001). Characterization and measurement of anthocyanins by UV-visible spectroscopy. Curr. Protoc. Food Anal. Chem..

[56] Tan C., Dadmohammadi Y., Lee M.C., Abbaspourrad A. (2021). Combination of Copigmentation and Encapsulation Strategies for the Synergistic Stabilization of Anthocyanins. Compr. Rev. Food Sci. Food Saf..

[57] Ross P.D., Subramanian S. (1981). Thermodynamics of Protein Association Reactions: Forces Contributing to Stability. Biochemistry.

[58] Oancea S. (2021). A review of the current knowledge of thermal stability of anthocyanins and approaches to their stabilization to heat. Antioxidants.

[59] Breiten B., Lockett M.R., Sherman W., Fujita S., Al-Sayah M., Lange H., Bowers C.M., Heroux A., Krilov G., Whitesides G.M. (2013). Water networks contribute to enthalpy/entropy compensation in protein–ligand binding. J. Am. Chem. Soc..

[60] Fisicaro E., Compari C., Braibanti A. (2004). Entropy/enthalpy compensation: Hydrophobic effect, micelles and protein complexes. Phys. Chem. Chem. Phys..

[61] Chen X., Gao Q., Liao S., Zou Y., Yan J., Li Q. (2022). Co-pigmentation mechanism and thermal reaction kinetics of mulberry anthocyanins with different phenolic acids. Foods.

[62] Patras A., Brunton N.P., O’Donnell C., Tiwari B.K. (2010). Effect of thermal processing on anthocyanin stability in foods; mechanisms and kinetics of degradation. Trends Food Sci. Technol..

[63] Boulton R. (2001). The copigmentation of anthocyanins and its role in the color of red wine: A critical review. Am. J. Enol. Vitic..

[64] Tang B., He Y., Liu J., Zhang J., Li J., Zhou J., Ye Y., Wang J., Wang X. (2019). Kinetic investigation into pH-dependent color of anthocyanin and its sensing performance. Dye. Pigment..

[65] Feng D., Jing P. (2025). pH-assisted and heat-induced nanostructures of beta-lactoglobulin for the stabilization of anthocyanins. Food Chem..

[66] Zhou N., Pan F., Ai X., Tuersuntuoheti T., Zhao L., Zhao L., Wang Y. (2022). Preparation, characterization and antioxidant activity of sinapic acid grafted chitosan and its application with casein as a nanoscale delivery system for black rice anthocyanins. Int. J. Biol. Macromol..

[67] Harbourne N., Jacquier J.C., Morgan D.J., Lyng J.G. (2008). Determination of the degradation kinetics of anthocyanins in a model juice system using isothermal and non-isothermal methods. Food Chem..

[68] Domínguez-López A., Remondetto G., Navarro-Galindo S. (2008). Thermal kinetic degradation of anthocyanins in a roselle (*Hibiscus sabdariffa* L. cv. ‘Criollo’) infusion. Int. J. Food Sci. Technol..

[69] Sendri N., Singh S., Sharma B., Purohit R., Bhandari P. (2023). Effect of co-pigments on anthocyanins of Rhododendron arboreum and insights into interaction mechanism. Food Chem..

[70] Zhao L., Pan F., Zhou N., Zhang Y.L., Hao S., Wang C.T. (2021). Screening of co-pigments to improve color stability of black rice anthocyanins and underlying mechanism. Food Sci..

[71] Nayak B., Berrios J.D.J., Powers J.R., Tang J. (2011). Thermal degradation of anthocyanins from purple potato (Cv. Purple Majesty) and impact on antioxidant capacity. J. Agric. Food Chem..

[72] Aprodu I., Milea Ș.A., Anghel R.M., Enachi E., Barbu V., Crăciunescu O., Râpeanu G., Bahrim G.E., Oancea A., Stănciuc N. (2019). New functional ingredients based on microencapsulation of aqueous anthocyanin-rich extracts derived from black rice (*Oryza sativa* L.). Molecules.

[73] Wu G., Yu L., Wu S., Li P., Wu C., Wang Y. (2025). Analysis of copigmentation between organic acids and cyanidin 3-*O*-glucoside in blackberry wine. Food Chem. X.

[74] Cortez R., Luna-Vital D.A., Margulis D., Gonzalez de Mejia E. (2017). Natural pigments: Stabilization methods of anthocyanins for food applications. Compr. Rev. Food Sci. Food Saf..

[75] Granados-Balbuena S.Y., Díaz-Pacheco A., García-Meza M.G., Tapia-López L., Cruz-Narváez Y., Ocaranza-Sánchez E. (2023). Phytochemical profile of petals from black *Dahlia pinnata* by flow injection analysis–electrospray ionization–Fourier transform ion cyclotron resonance mass spectrometry. Phytochem. Anal..

[76] Singleton V.L., Rossi J.A. (1965). Colorimetry of total phenolics with phosphomolybdic-phosphotungstic acid reagents. Am. J. Enol. Vitic..

[77] Brand-Williams W., Cuvelier M.E., Berset C. (1995). Use of a free radical method to evaluate antioxidant activity. LWT-Food Sci. Technol..

